# A Novel Her2/VEGFR2/CD3 trispecific antibody with an optimal structural design showed improved T-cell-redirecting antitumor efficacy

**DOI:** 10.7150/thno.75037

**Published:** 2022-11-14

**Authors:** Dong Liu, Xuexiu Qi, Xiaoyi Wei, Lijun Zhao, Xuechun Wang, Shuhong Li, Zhidong Wang, Licai Shi, Jiean Xu, Mei Hong, Zhong Liu, Lili Zhao, Xiankun Wang, Bo Zhang, Yuhan Zhang, Feng Wang, Yu J. Cao

**Affiliations:** 1 State Key Laboratory of Chemical Oncogenomics, Guangdong Provincial Key Laboratory of Chemical Genomics, Peking University Shenzhen Graduate School, Shenzhen, Guangdong, 518055, China.; 2 Lunan Pharmaceutical Group Co., Ltd, Feixian County, Shandong, 273400, China.; 3 National Engineering Laboratory of High Level Expression in Mammalian Cells, Feixian County, Shandong, 273400, China.; 4 Institute of Neurological and Psychiatric Disorders, Shenzhen Bay Laboratory, Shenzhen, Guangdong, 518132, China.; 5 Key Laboratory of Protein and Peptide Pharmaceuticals, Beijing Translational Center for Biopharmaceuticals Institute of Biophysics, Chinese Academy of Sciences Beijing 100101, China.; 6 Institute of Chemical Biology, Shenzhen Bay Laboratory, Shenzhen, 518132, China.

**Keywords:** Trispecific Antibodies, Protein Engineering, Site-specific Recombination, Immunological Synapse, Therapy Resistance

## Abstract

**Rationale:** T-cell-redirecting bispecific antibodies (bsAbs) and trispecific antibodies (tsAbs) designed to recognize different epitopes or antigens have emerged as promising cancer therapies. Current approaches are all designed to include another antibody specific to the site of the primary antibody, and the molecular structures are generally established. However, the dimensions of target molecule and epitope location play a key role in the efficiency of the immunological synapse (IS) formation and subsequent T-cell-redirecting activities, therefore the connection flexibility of these antibodies determines the geometries of different formats of these molecules and will have a major impact on the efficacy.

**Methods:** We describe a novel recombination strategy using various linker designs to site-specifically fuse anti-Her2 (2Rs15) or anti-VEGFR2 (3VGR19) nanobodies to different positions of the anti-CD3 antibody fragment (Fab, SP34). Based on the comparison among the various antigen-specific bsAbs, we could determine the desired fusion site of each nanobody to SP34, and further ensure the optimal structure of tsAbs with synergistic dual-antigen enhanced T-cell-redirecting activities.

**Results:** This approach allows precise control of the formation of IS between Her2- and/or VEGFR2-expressing cancer cells and T cells, to obtain the optimal structure of the Her2/VEGFR2/CD3 tsAb without the need to map antibody-binding epitopes. Optimization of Her2/VEGFR2/CD3 tsAb results in enhanced T-cell-redirecting *in vitro* and *in vivo* antitumor efficacy compared with the corresponding bsAbs alone or in combination, and the potency to overcome tumor relapse due to antigen escape or resistance to Herceptin and Cyramza therapy.

**Conclusion:** The novel design strategy for developing tsAbs using a site-specific recombination approach represents a promising platform for immuno-oncology and in applications other than cancer therapy.

## Introduction

Cancer immunotherapy, which harnesses the power of the immune system to treat tumors has emerged as a remarkable new therapy for patients with cancer 1, 2. Based on clinical promise, interest in T-cell redirection strategies for cancer therapy, particularly those involving the use of bispecific antibodies (bsAbs) such as bispecific T-cell engagers (BiTEs), which trigger anticancer immunity, has substantially increased 3, 4. Indeed, several clinical successes of bsAb therapies have been reported; among the eight approved bsAbs, Blincyto (blinatumomab), Removab (catumaxomab), Kimmtrak (tebentafusp-tebn), Mosunetuzumab (Lunsumio) and Teclistamab (Tecvayli) which redirect T-cell specificity, have dominated the novel cancer therapies developed through scientific research and clinical studies 5-9. However, these bsAbs, endowed with one antigen binding to CD3 on T cells and the other antigen on tumor cells, may drive the outgrowth of tumor antigen escape variants 10, 11. This example of solid tumors is more problematic when targeting tumor-associated antigens (TAAs), where antigen expression is generally less homogenous than hematological markers.

One strategy to overcome the outgrowth of antigen-deficient tumor cells is to equip T cells with two targeting moieties to different TAAs, as has been substantially documented in chimeric antigen receptor-T (CAR-T) cell therapy 12, 13. T cells simultaneously engineered with CAR to recognize dual antigens have been successfully applied to redirect various hematological and solid malignancies 14, 15. Preclinical studies have shown that this approach results in increased antitumor function compared with that obtained with CAR-T cells specific for one TAA 16, 17. Although a few studies explored trispecific antibodies (tsAbs) with the feasibility of eliciting an anticancer response for dual-TAA targeting 18-21, researchers have not yet determined whether the constructs were well designed based on the molecular events relating to optimal bsAb configuration.

T-cell-mediated bsAbs are known to facilitate the formation of a cytolytic immune synapse (IS) by simultaneously binding to the TCR subunit CD3ε on T cells and the TAA on the target cell 22. In the IS formed by the native TCR, the distance between the T cell and antigen-presenting cell is reportedly 130~150 Å, which is important for sterically excluding inhibitory phosphatases such as CD45 and CD148 from the IS 23, 24. Based on bsAbs and CAR-T cells, we and other groups have reported precise control of the geometry and stoichiometry of IS formation by switch molecules requiring optimization of the different epitope areas recognized by redirected antibodies 25-27. Therefore, the generation of tsAbs that are readily endowed with optimal individual TAA-associated IS formation is highly desirable to minimize the risk of developing escape variants and to simplify the clinical implementation of T-cell-redirecting therapy.

**Scheme 1:**
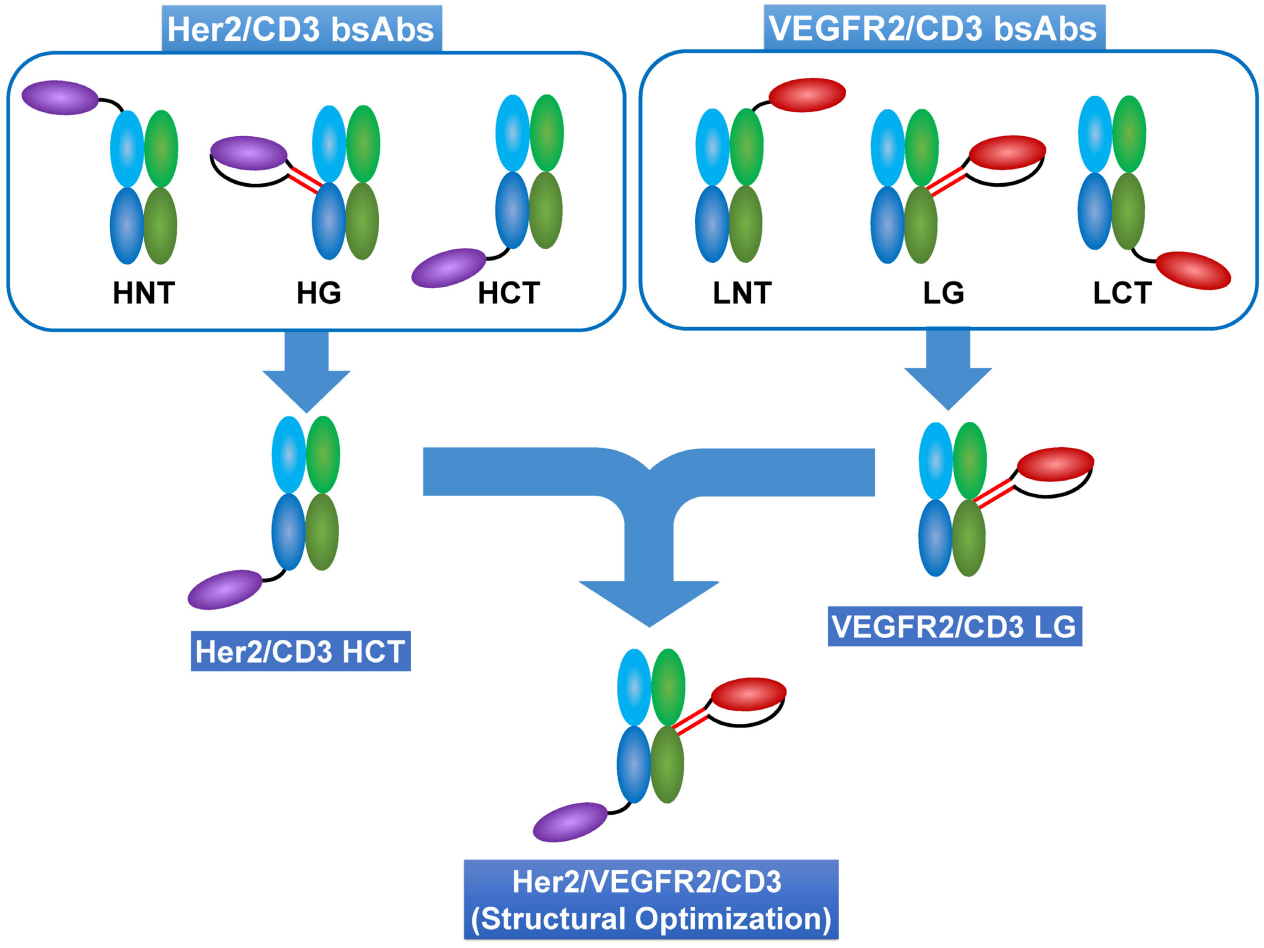
** Strategy for optimal structural design of tsAbs.** Using a site-specific recombination strategy, anti-Her2 (2Rs15d) or anti-VEGFR2 (3VGR19) nanobodies were fused to anti-CD3 Fab (SP34) at different positions to generate various formats of Her2/CD3 and VEGFR2/CD3 bsAbs. HNT: 2Rs15d fused to the N-terminus of SP34 HC; HG: 2Rs15d fused to S184-L187 of SP34 HC; HCT: 2Rs15d fused to the C-terminus of SP34 HC; LNT: 3VGR19 fused to the N-terminus of SP34 LC; LG: 3VGR19 fused to S171-D173 of SP34 LC; LCT: 3VGR19 fused to the C-terminus of SP34 LC. Theoretically, different formats of bsAbs could control distinct geometries resulting from IS formation between target cells and T cells. Therefore, based on the comparison among the TAA-specific bsAbs, we could determine the desired fusion site of each nanobody to SP34 Fab. This finding will shed light on the optimal structure of tsAbs considering the synergistic dual antigen-enhanced T-cell-redirecting activity.

To design a unique format with optimal T-cell-redirecting activity toward either the Her2 or VEGFR2 on cancer cells, we modified the recombination approach using various linker designs for the site-specific fusion of Her2 (2Rs15)- or VEGFR2 (3VGR19)- specific nanobodies to various sites of the CD3 Fab (SP34). This recognized method for bsAb design produces a variety of structural options and thus results in the different scenarios in which an artificial IS forms when mimicking physiological membrane spanning or does not efficiently form when distal or close epitope binding is applied. Consequently, this phenomenon allows for increased efficiency in realizing the optimal structure of tsAbs showing synergistic dual antigen-enhanced T-cell-redirecting activity (Scheme 1).

## Results

### Structural determination of T-cell-redirecting bsAbs against Her2 or VEGFR2

**Figure 1:**
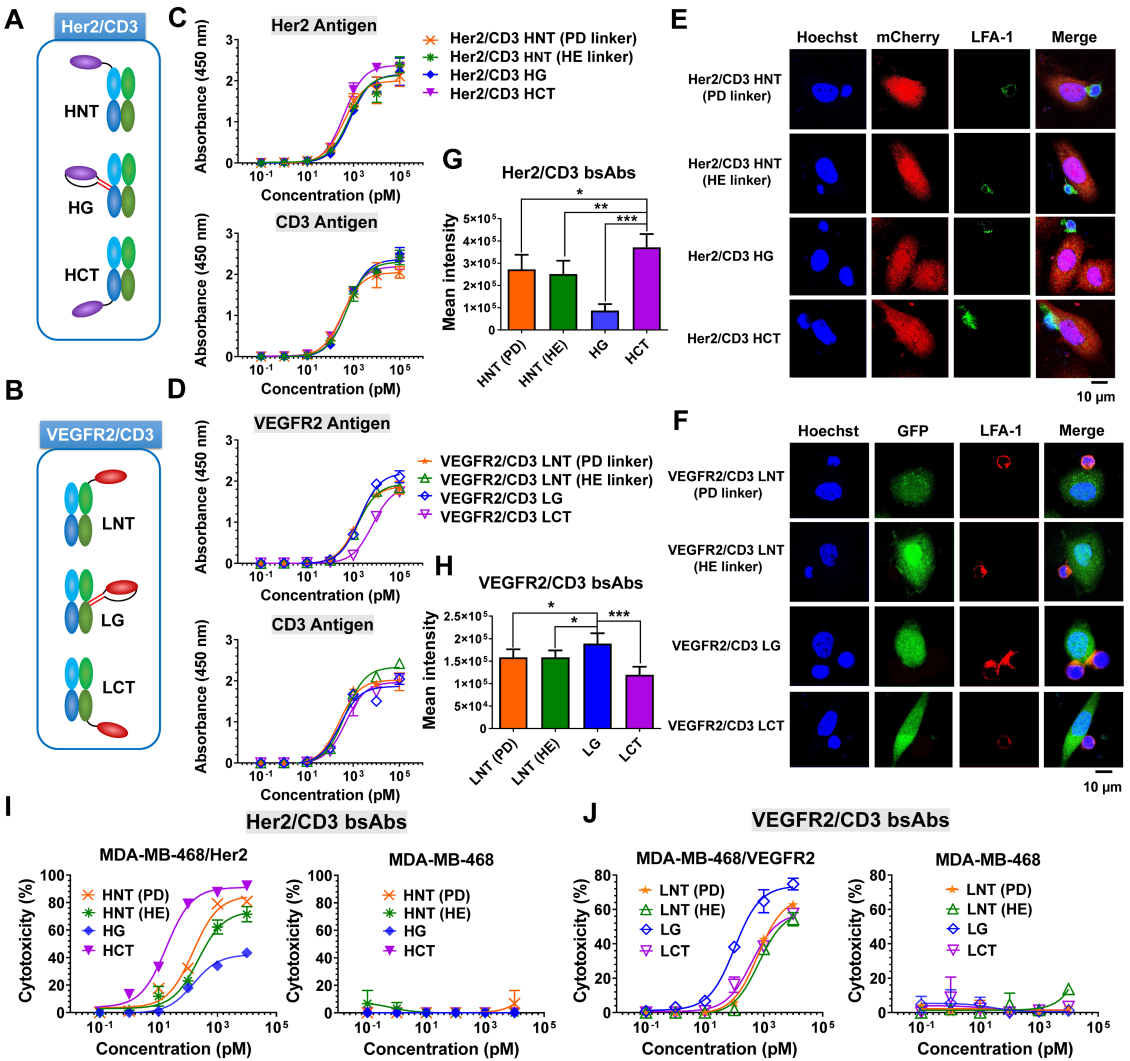
** Development and characterization of Her2/CD3 and VEGFR2/CD3 bsAbs. (A)** and **(B)** Schematic structures of Her2/CD3 and VEGFR2/CD3 bsAbs. **(C)** and **(D)** Binding activities of different Her2/CD3 and VEGFR2/CD3 bsAbs to human Her2-, VEGFR2- or CD3-extracellular domains by ELISA. **(E)** and **(F)** Confocal immunofluorescent observation of IS formation triggered by different Her2/CD3 and VEGFR2/CD3 bsAbs between Her2- or VEGFR2-transduced MDA-MB-468 and Jurkat cells. LFA-1 was labelled to show IS formation. **(G)** and **(H)** Quantitative comparison of the cell contact area based on the area of LFA-1 accumulation in the IS (mean ± SD, n=9). Asterisks indicate statistical significance using the Newman Keuls multiple comparison test. *P<0.05; **P <0.01; ***P<0.001.** (I)** and **(J)** Cytotoxicity of T cells against target cells mediated by titration of different Her2/CD3 or VEGFR2/CD3 bsAbs. The cytotoxicity assays were performed with an E: T ratio of = 20: 1 and after 24 h incubation. Cytotoxicity was assessed by LDH release.

To fulfill the optimal geometry between target cells and T cells, three formats of Her2/CD3 or VEGFR2/CD3 bsAbs were designed by site-specifically fusing a Her2-specific nanobody (2Rs15d) or VEGFR2-targeted nanobody (3VGR19) to the defined sites of the HC or LC of CD3-targeted antibody Fab (SP34): Her2/CD3 HNT (2Rs15d fused to the N-terminus of SP34 HC), Her2/CD3 HG (2Rs15d fused to S184-L187 of SP34 HC), Her2/CD3 HCT (2Rs15d fused to the C-terminus of SP34 HC) (Figure 1A and Figure S1), VEGFR2/CD3 LNT (3VGR19 fused to the N-terminus of SP34 LC), VEGFR2/CD3 LG (3VGR19 fused to S171-D173 of SP34 LC), VEGFR2/CD3 LCT (3VGR19 fused to the C-terminus of SP34 LC) (Figure 1B and Figure S2). As a method to fully display nanobodies on Fab without affecting the binding activities, we applied a rigid peptide linker from pyruvate dehydrogenase (PD linker) or α-helical EAAAK linker (HE linker) to expand the nanobody to the N-terminus of the HC or LC of SP34 Fab or flexible GGGGS linker to fuse the nanobody to the C-terminus of the HC or LC of Fab. In addition, the nanobody was site-specifically grafted to the designated sites of the HC of SP34 Fab with antiparallel coiled-coil stalk linkers 28. All linker sequences are listed in Table S1. The fusion constructs of either HC or LC were expressed in FreeStyle HEK293 cells by transient transfection and purified using Protein G chromatography, followed by size-exclusion chromatography to remove the aggregates. Each bsAb was then analyzed by SDS-PAGE (Figure S3-S4) and gel filtration (Superdex 200) analysis (Figure S5-S6), and the results revealed the expected molecular weight and an overall purity of > 95%.

To characterize various bsAb designs, we tested a panel of cell lines with Her2 or VEGFR2 expression (Figure S7), and generated Her2- and/or VEGFR2-transduced MDA-MB-468 cell lines (Figure S8). The binding ability of different forms of Her2/CD3 or VEGFR2/CD3 bsAbs was verified by an ELISA using immobilized Her2, VEGFR2 or CD3 antigen. As shown in Figure 1C, all the Her2/CD3 bsAbs showed overall comparable apparent binding affinities to Her2 and CD3. In contrast, the binding affinity of VEGFR2/CD3 LCT was 5-fold lower for VEGFR2 than that of other VEGFR2/CD3 constructs (Figure 1D), however, all VEGFR2/CD3 constructs exhibited similar binding profiles for CD3. Flow cytometry assays were subsequently performed to show that all the bsAb constructs produced staining intensities with CD3-positive Jurkat cells, confirming virtually identical binding activities (Figure S9).

The formation of an effector cell IS in cytotoxic T lymphocytes is a hierarchical and stepwise rearrangement of structural and signaling components and involves the targeted release of the contents of lytic granules 22, 24. To verify the IS formation efficiency between T cells and tumor cells triggered by different bsAb formats, we examined the engagement of the adhesion molecule LFA-1, which is involved in IS formation, using laser-scanning confocal microscopy. As shown in Figure 1E-F, after a 4-h incubation, we observed greater LFA-1-mediated polarization of Jurkat cells to Her2-transduced MDA-MB-468 cells in the presence of Her2/CD3 HCT compared with the other Her2/CD3 formats, and higher LFA-1 polarization efficiency between Jurkat cells and MDA-MB-468/VEGFR2 cells treated with VEGFR2/CD3 LG compared with other VEGFR2/CD3 candidates. The significant differences in LFA-1 enrichment in the cytolytic IS were mediated by the different bsAb formats (Figure 1G-H), which suggested that distinct geometries were needed to achieve optimal synapse formation with each antigen-antibody interaction. The efficiency was statistically assessed and reflected the ability of Her2/CD3 HCT and VEGFR2/CD3 LG to induce sufficient TCR signaling mediated by human T cells compared with other bsAb partners. Using an imaging approach, the bsAbs were readily optimized using a unique site-specific recombination strategy to obtain highly effective geometries for T cell and target cell interactions, regardless of the mapping of antigen-antibody pairing. Specificity for T-cell activation is a critical design consideration when evaluating bsAb-based T-cell-redirecting cytotoxicity. After 24 h of incubation, we observed significantly higher expression of CD25 on CD8+ cytotoxic T cells treated with Her2/CD3 HCT and VEGFR2/CD3 LG than in the groups treated with the other bsAbs (Figure S10-S11). We then evaluated the ability to induce T-cell effector functions, and determined that the differences in cytotoxicity among various bsAb formats were related to the T cell activation efficiency. As shown in Figure 1I, Her2/CD3 HCT (IC_50_ = 18.33 pM) was more potent than other Her2/CD3 bsAbs (IC_50_ = 147.91, 221.50 and 151.15 pM for HNT (PD linker), HNT (HE linker) and HG, respectively) against MDA-MB-468/Her2 cells. This enhancement was also confirmed in Her2-positive MDA-MB-435 and PC3 tumor cells (Figure S12). Comparison of the VEGFR2/CD3 bsAbs showed that VEGFR2/CD3 LG (IC_50_ = 101.91 pM) displayed the overall best specific toxicity compared with LNT (PD linker, IC_50_ = 626.42 pM), LNT (HE linker, IC_50_ = 678.60 pM) and LCT (IC_50_ = 389.91 pM) toward MDA-MB-468/VEGFR2 cells (Figure 1J), and we confirmed the observed differences in another VEGFR2-positive human umbilical vein endothelial cell (HUVEC) line (Figure S13). In addition to lytic activity, all the bsAbs induced IL-2, IFN-γ and TNF-α release that correlated with the degree of cytotoxicity, and Her2/CD3 HCT and VEGFR2/CD3 LG showed the highest cytokine production (Figure S14-S15). Overall, the ability to maneuver the incorporation site of Her2- or VEGFR2-specific nanobodies into the CD3 Fab significantly alters IS formation between effector and target cells, resulting in differences in T-cell activation, cytokine release and T-cell-dependent cytotoxic activity.

### Structural design of Her2/VEGFR2/CD3 tsAb

**Figure 2:**
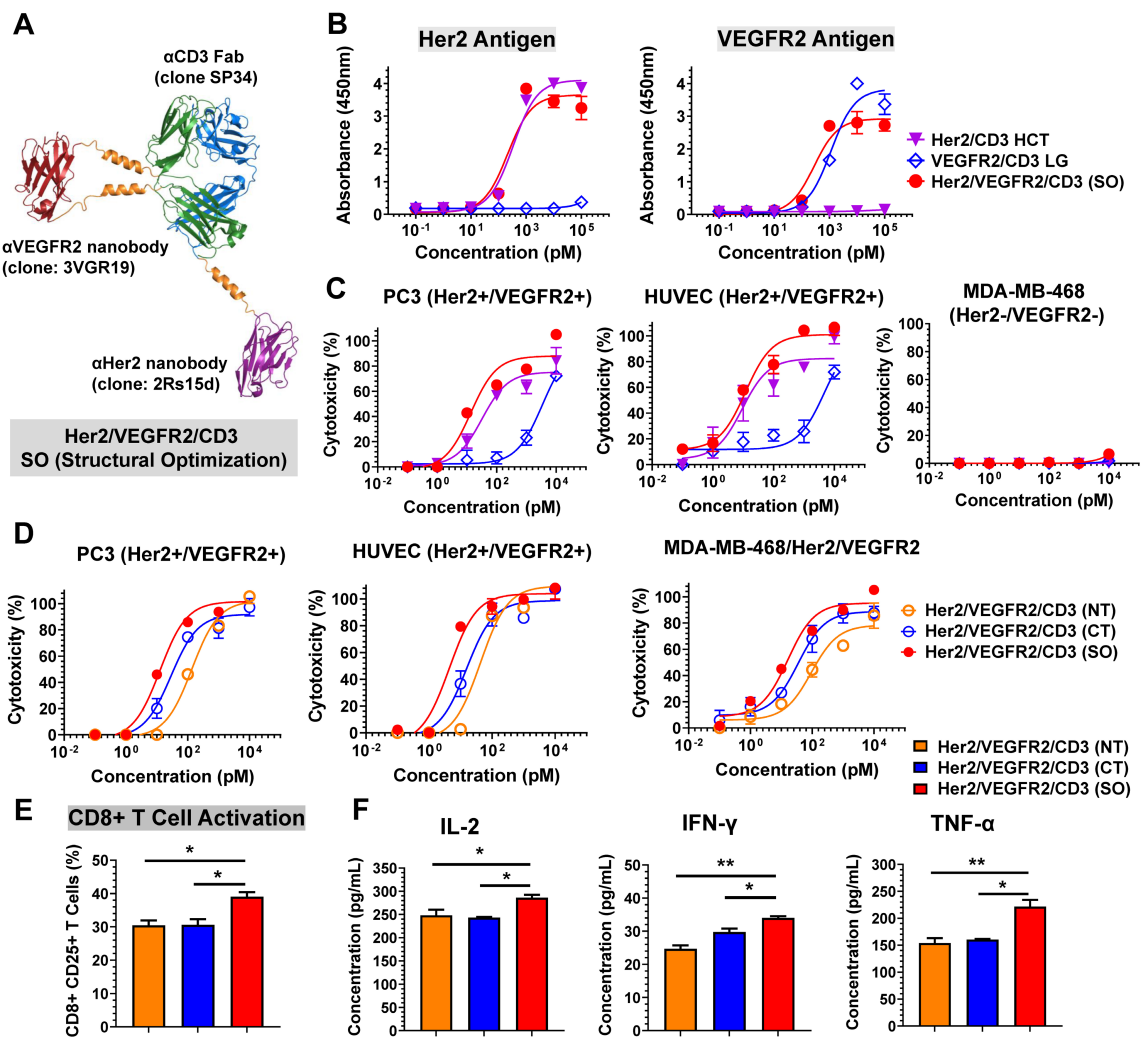
** Design and characterization of Her2/VEGFR2/CD3 (SO). (A)** Ribbon diagrams depicting the tsAb obtained by structural design optimization: the HC of SP34 shown in blue was fused with anti-Her2 nanobody (2Rs15) in purple at its C-terminus, and the LC in green was grafted with anti-VEGFR2 nanobody (3VGR19) in red with a rigid linker. **(B)** Profiles of the binding of Her2/VEGFR2/CD3 (SO) and the corresponding Her2/CD3 HCT and VEGFR2/CD3 LG bsAbs to Her2 and VEGFR2 antigens determined by ELISA. **(C)** Cytotoxicity assays comparing Her2/VEGFR2/CD3 (SO) with the bsAbs against PC3 cells, HUVECs and MDA-MB-468 cells. **(D)** Cytotoxicity of different forms of Her2/VEGFR2/CD3 tsAbs redirecting T cells against Her2/VEGFR2-positive cancer cells. The cytotoxicity assays were performed with an E: T ratio of = 10: 1 and after 24 h incubation. The cytotoxicity was assessed by LDH release. The data are presented as the means ± SDs. **(E)** Activation of CD8+ T cells by PC3 cells in the presence of different tsAb formats. T cells were measured by anti-CD8/anti-CD25 antibody cocktails by flow cytometry. **(F)** IL-2, TNF-α and IFN-γ release from T cells cultured with PC3 cells in the presence of tsAbs at 100 pM. The data are presented as means ± SDs, and statistical significance was calculated using the Newman Keuls multiple comparison test. *P<0.05; **P <0.01; ***P<0.001.

Based on the structural designs of Her2/CD3 and VEGFR2/CD3 bsAbs, we developed Her2/VEGFR2/CD3 (SO) by fusing the 2Rs15d to the C-terminus of SP34 HC and grafting the 3VGR19 to the S171-D173 of LC (Figure 2A). Following the expression and purification steps mentioned above, we confirmed the purity and molecular weight by SDS-PAGE (Figure S16) and gel filtration analysis (Figure S17). As shown in Figure 2B, we assessed the binding affinity of Her2/VEGFR2/CD3 (SO) to immobilized antigen by ELISA and found the similar binding profiles of tsAb as their corresponding bsAb partners. To evaluate the ability of tsAb to redirect T cells against Her2+/VEGFR2+ cancer cells, we performed a cytotoxicity assay comparing the bsAb candidates. As shown in Figure 2C and Figure S18, Her2/VEGFR2/CD3 (SO) exhibited an overall improved killing activity compared with Her2/CD3 HCT and VEGFR2/CD3 LG against Her2+/VEGFR2+ PC3, HUVEC, AsPC-1 and MCF-7 cells. Significant differences in CD8+ T-cell activation (Figure S19) and cytokine production (IL-2 and TNF-α) by T cells (Figure S20) were also confirmed with structural optimized tsAb against PC3 cells. Further comparison among single- or dual-target tumor cells confirmed that the enhanced avidity effects required both target antigens recognized and mediated by Her2/VEGFR2/CD3 (SO) against MDA-MB-468/Her/VEGFR2 (IC_50_ = 1.06 pM) in comparison to MDA-MB-468/Her2 (IC_50_ = 15.85 pM) and MDA-MB-468/VEGFR2 (IC_50_ = 371.54 pM) (Figure S21).

Subsequently, we prepared two other tsAb formats, Her2/VEGFR2/CD3 (NT, fusing each nanobody to the N-terminus of SP34) and Her2/VEGFR2/CD3 (CT, fusing each nanobody to the C-terminus of SP34), as comparable candidates. Following purity analysis by size exclusion chromatography (Figure S22), the binding of each tsAb to its antigen was determined by ELISA. As shown in Figure S23, all the constructs exhibited similar apparent binding affinities to Her2 and CD3 antigens, but Her2/VEGFR2/CD3 (CT) showed reduced affinities to VEGFR2 compared to the other candidates. Further comparison indicated that Her2/VEGFR2/CD3 (SO) was superior in inducing T-cell-specific cytotoxicity against PC3 cells, HUVECs and MDA-MB-468/Her2/VEGFR2 cells compared with the tsAbs obtained by fusing nanobodies to both the N- or C-terminus of the Fab (Figure 2D). In addition, the assessment of CD25 upregulation in CD8+ T cells (Figure 2E) and inflammatory cytokine production (Figure 2F) confirmed that Her2/VEGFR2/CD3 (SO) was the best structural configuration for antigen-dependent T-cell activation. These results clearly demonstrate that tsAbs can be structurally optimized simply based on the corresponding bsAb partner to facilitate formation of the optimal IS geometry between effector and target cells and directly affect T-cell-redirecting activity. Further stability analysis of Her2/VEGFR2/CD3 (SO) revealed that no reduction in the binding activity or specific cytotoxicity of tsAb was detected after 72 h of incubation in the presence of human plasma (Figure S24-S25), suggesting that the unusual structure of tsAb was quite stable in human plasma and may guarantee its *in vivo* potency in therapeutic applications.

### Enhanced *in vivo* antitumor efficacy of tsAb

**Figure 3:**
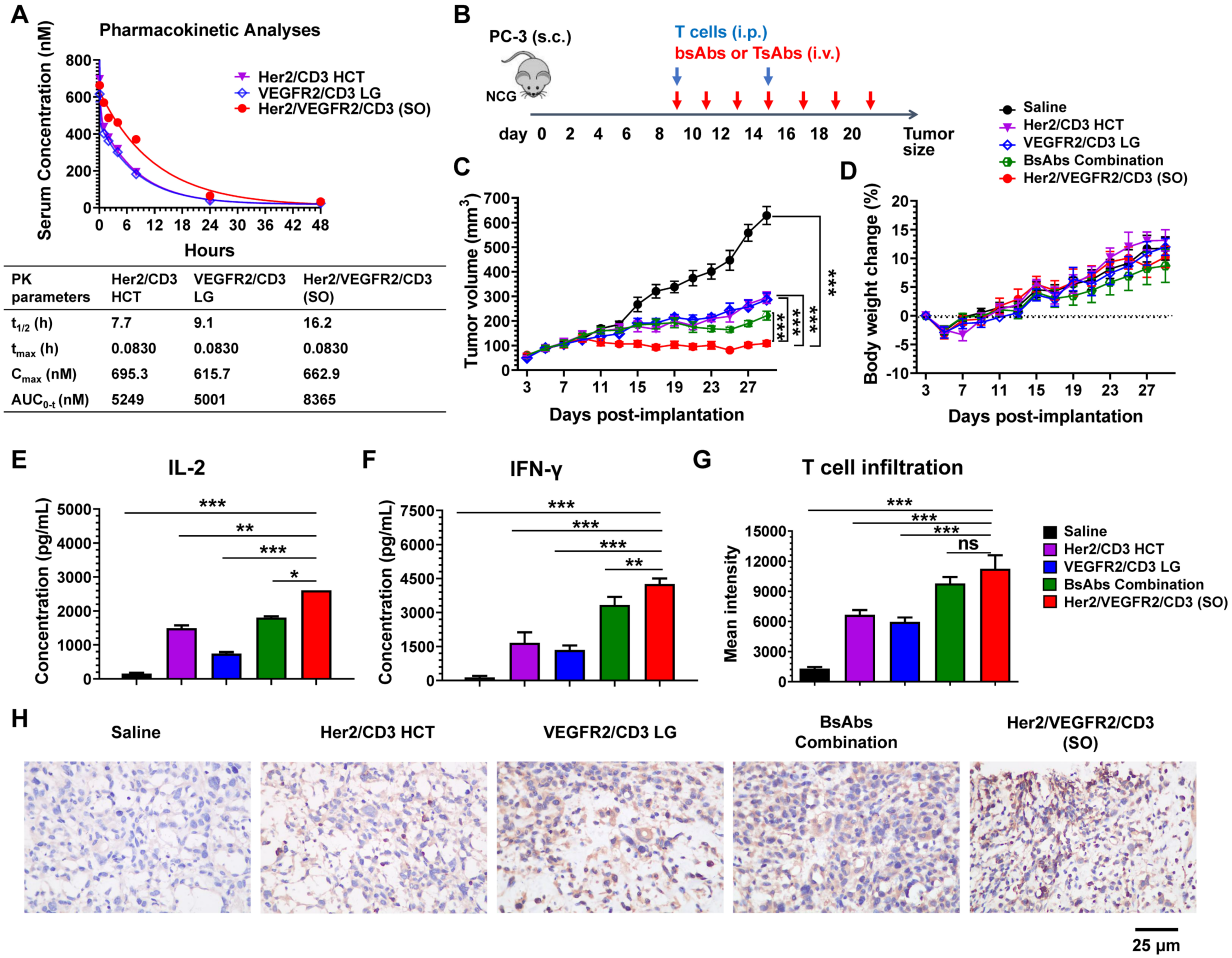
** PK and *in vivo* efficacy comparison of bsAbs and tsAb. (A)** PK of Her2/VEGFR2/CD3 (SO) and the corresponding bsAbs (Her2/CD3 HCT and VEGFR2/CD3 LG) in mice. The bsAbs and tsAb were injected i.v. into mice at 15 nmol/kg (n=3/group), and serum was isolated for determination of the antibody concentration. Concentration vs. time curves were evaluated by noncompartmental analysis using WinNonlin. The values shown are the average from 3 mice per the group. t1/2, half-life; tmax, time of maximum concentration; Cmax, maximum concentration; AUC0→inf, area under the concentration-time curve extrapolated to infinity. **(B)** Schematic representation of the study design and timeline of the treatment. A total of 2×10^6^ PC3 cells in 50% Matrigel were s.c. implanted into 6- to 8-week-old male NCG mice. Nine days later, the mice were i.p. infused twice with 25×10^6^ activated T cells every five days, and bsAb or tsAb treatment was administered i.v. at 15 nmol/kg or saline every other day for a total of seven doses. **(C)** Tumor burden and **(D)** body weight were monitored twice weekly. The mean tumor volume was calculated as W × L × H, which was measured with digital calipers. Each data point represents the mean from 5 mice per group ± SD. **(E)** and **(F)** Quantification of the IL-2 and IFN-γ levels 24 h after first the antibody dose (n=5). **(G)** Quantification of human T-cell infiltration in the tumor area based on the immunofluorescent staining analysis. The results are shown as means ± SDs (n=15). Statistical significance was calculated using the Newman Keuls multiple comparison test (C, E, F, H and I): *P < 0.05, **P < 0.01, and ***P < 0.001; ns, not significant (≥0.05). **(H)** Immunohistochemistry analysis of human granzyme B expression in tumor tissues after antibody treatment.

To determine whether the activities observed in the *in vitro* assays translate to *in vivo* mouse xenograft models, we first evaluated the pharmacokinetics (PK) of the bsAbs (Her2/CD3 HCT or VEGFR2/CD3 LG) and tsAb (Her2/VEGFR2/CD3 (SO)) after i.v. injection into female BALB/c mice. The PK of each drug in mice was determined by measuring the antibody levels in serum samples collected at various time points. As shown in Figure 3A, both bsAbs showed similar PK profiles with terminal half-lives ranging from 7.7 to 9.1 h, similar to the results obtained in previous studies of BiFab 29. However, the clearance of Her2/VEGFR2/CD3 (SO) from the circulation was prolonged compared with that of the bsAbs, with a half-life of 16.2 h, which might be attributed to the increased overall molecular weight of the tsAb.

We further tested the *in vivo* efficacy of the tsAb and bsAbs in mouse xenograft models using PC3 prostate tumor xenografts. Briefly, tumors were s.c. inoculated in NCG mice to allow the formation of palpable solid tumors. Treatment was initiated by an i.p. infusion of human T cells and continued by i.v. injections of the bsAbs or their combination or the tsAb every other day (Figure 3B). As shown in Figure 3C, tumor regression was observed in mice administered either the bsAbs or the tsAb, whereas tsAb treatment showed significantly durable tumor growth inhibition compared with saline, each bsAb alone or their combination. In addition, no drug-induced toxicity was observed in the mice treated in this experiment (Figure 3D). We next examined whether the cytokine release or T-cell infiltration in the tumor microenvironment correlated with the tumor growth inhibition observed after treatment with each drug. As shown in Figure 3E-F, the levels of inflammatory cytokines (human IL-2 and IFN-γ) in the animals dosed with Her2/VEGFR2/CD3 (SO) were higher than those in the animals that received the other drugs, but no detectable human or murine IL-6 was observed in these mice (Figure S26), suggesting that the tsAb could efficiently induce tumor-redirected T-cell activation, but was potentially safe in avoiding cytokine storm syndrome in cancer patients. We also observed increased human T-cell infiltration (human CD3) in the PC3 tumor area treated with the tsAb, compared with the groups dosed with saline or bsAbs (Figure S27). Quantification confirmed that the optimally designed Her2/VEGFR2/CD3 (SO) significantly increased T-cell recruitment in the tumor microenvironment compared with saline and bsAbs, but this significance was not found between tsAb and the bsAbs combination (Figure 3G), which suggested that the tumor suppression with tsAb treatment could be in part due to the synergistic T-cell-redirecting activation in response to dual antigen expression on tumors. Further immunohistochemistry staining results confirmed that tsAb was able to efficiently redirect T cells to infiltrate tumor area and trigger T-cell signalling and cytotoxic granzyme B expression (Figure 3H). We did not observe antigen loss or downregulation in PC3 tumors by these antibody fusions (Figure S28-S29), which may suggest that recurrence by bsAbs was probably associated with inefficient T-cell-redirecting activities in response to tumors with low antigen densities, a challenge that may be addressed by tsAb designs to enhance the avidity effects.

### Efficacy of the tsAb against tumors resistant to Herceptin and Cyramza

Acquired resistance to conventional antibodies is common in patients who have previously been treated with Herceptin, Cyramza or their combination 30. In this study, we developed a model of Herceptin and Cyramza resistant variants of PC3 cells. As shown in Figure S30, parental PC3 cells were readily sensitive to Herceptin (IC_50_ = 0.32 nM), Cyramza (IC_50_ = 1.91 nM) and their combination (IC_50_ = 0.93 nM), but the resistant cells did not show growth sensitivity to these antibodies. Further analysis revealed no differences in Her2- and VEGFR2- expression between the parental and resistant PC3 cells (Figure S31). Specifically, treatment of these resistant cells did not result in cross-resistance to Her2/VEGFR2/CD3 (SO) compared with the results found for the parental cells (IC_50_ = 11.42 and 11.81 pM for the parental and resistant cells, respectively).

**Figure 4:**
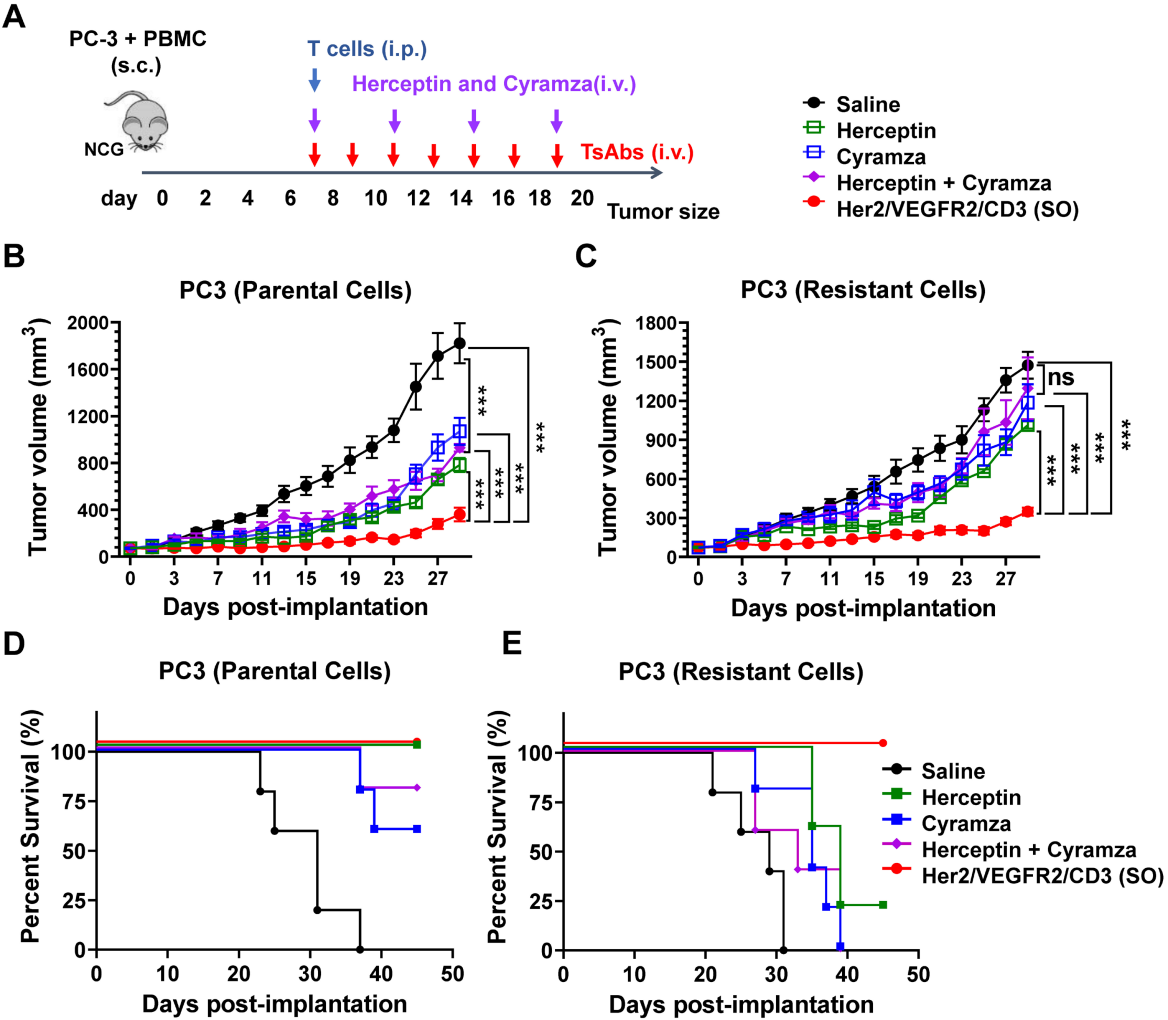
**Efficacy study of Her2/VEGFR2/CD3 (SO) against PC3 tumors resistant to Herceptin and Cyramza. (A)** Schematic representation of the *in vivo* study design and timeline of the treatment. Seven days after s.c. implantation of the mixture of 2×10^6^ PC3 (parental or resistant) cells and PBMCs in 50% Matrigel, the mice were i.p. injected with 20×10^6^ activated T cells and then i.v. administered 15 nmol/kg of Herceptin, Cyramza or the combination four times every three days or the tsAb seven times every other day. **(B)** and **(C)** Growth of the parental and resistant PC3 tumors treated with antibodies or the tsAb was monitored and compared twice weekly. Data are presented as means ± SDs, and statistical significance was calculated using the Newman Keuls multiple comparison test: *P < 0.05, **P < 0.01, and ***P < 0.001; ns, not significant (≥0.05).** (D)** and** (E)** Survival curve of mice bearing parental and resistant PC3 tumors treated with antibodies or tsAb.

The *in vivo* efficacy of tsAb and antibodies (Herceptin, Cyramza, or their combination) was further compared in both PC3 tumor models. As shown in Figure 4A, xenograft models were established by s.c. implantation of a mixture of PC3 tumor cells (parental or resistant) and human peripheral blood mononuclear cells (PBMCs) into NCG mice. Upon formation of a palpable tumor, the mice were infused with expanded human T cells into the peritoneal cavity. Based on their different half-lives, the mice were i.v. administered 15 nmol/kg/dose of the antibodies (every three days, four times) or tsAb (every other day, eight times). As shown in Figure 4B, the mice in the parental PC3 tumor group treated with either the antibodies or tsAb exhibited persistent tumor growth inhibition compared with the mice that only received T cells and saline. In the resistant tumor model (Figure 4C), although the dosing regimen was the same, the mice treated with tsAb showed a significant tumor growth delay compared with the control mice, whereas conventional antibodies were marginally effective. Mouse survival was monitored in both the parental and resistant PC3 models, and the mice treated with tsAb had longer survival times than those treated with the antibodies (Figure 4D-E). The tumor tissue was further immunofluorescently stained to analyze cell death using the TUNEL assay and proliferation using Ki67 staining. As shown in Figure S32-S33, tumor sections from mice treated with tsAb showed a considerably greater number of apoptotic nuclei and substantially lower Ki67 staining in PC3 parental and resistant xenograft models than those from mice treated with saline or antibodies. Significant differences in the intensity of TUNEL- and Ki67-positive cells were further confirmed using the Newman Keuls multiple comparison test (Figure S34-S35). A histological examination of vital organs for subacute toxicity, including the heart, kidney, liver, lung and spleen (Figure S36-S37), did not reveal any gross pathological lesions during the treatment.

### Potential activity of the tsAb in avoiding immune evasion

**Figure 5:**
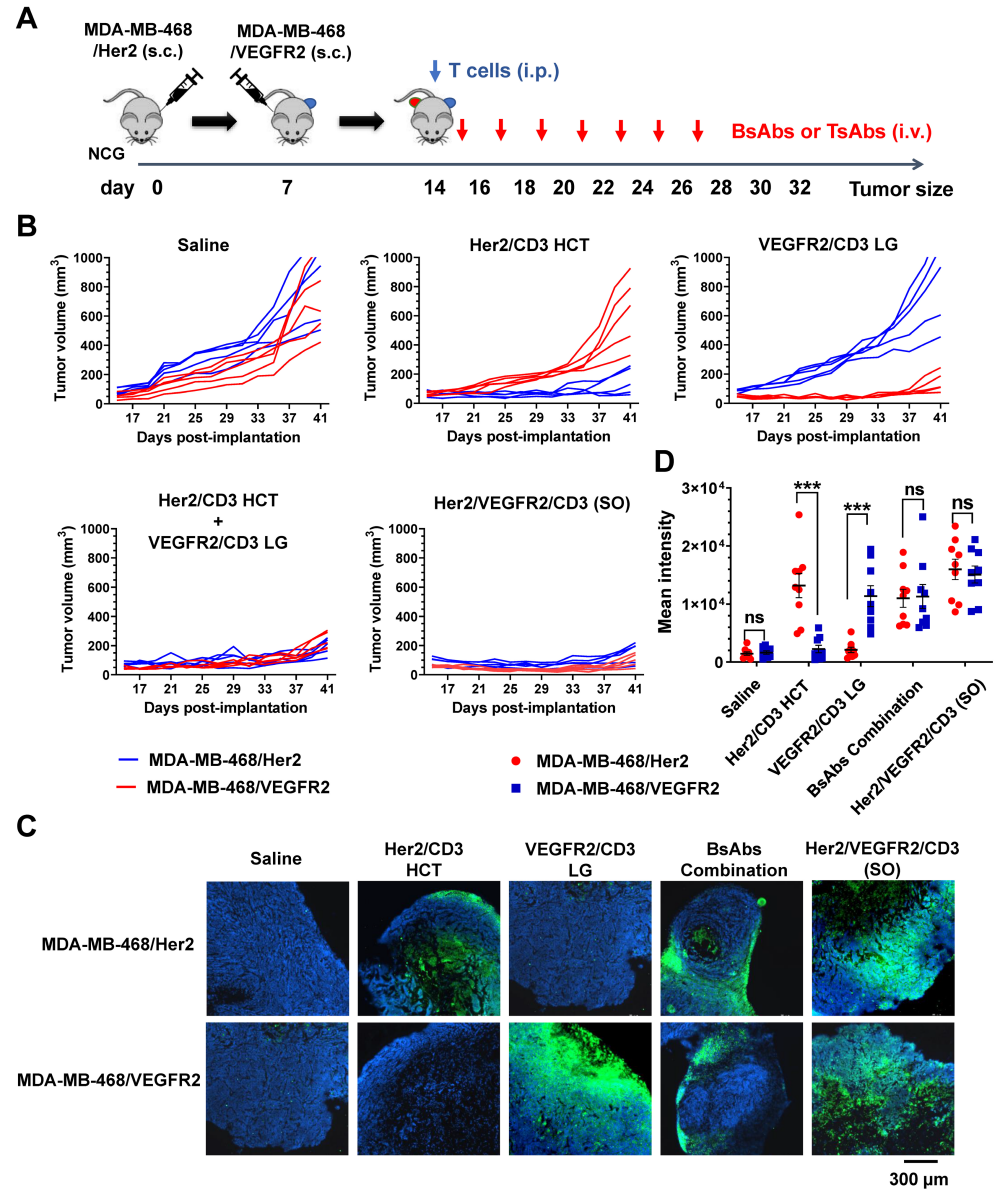
**Study of the efficacy of Her2/VEGFR2/CD3 (SO) in overcoming immune evasion. (A)** Schematic representation of the study design and timeline of the treatment. NCG mice were implanted with 5×10^6^ MDA-MB-468/Her2 cells in the left flank on day 0. On day 7, the mice received 10×10^6^ MDA-MB-468/VEGFR2 cells in the right flank. On day 14, the mice were i.p. infused with T cells and i.v. treated with 15 nmol/kg Her2/VEGFR2/CD3 (SO) or bsAbs (Her2/CD3 HCT, VEGFR2/CD3 LG or their combination) seven times every other day. **(B)** MDA-MB-468/Her2 (red) and MDA-MB-468/VEGFR2 (blue) growth kinetics of tumor-bearing mice that received saline, bsAb alone or in combination, or tsAb. **(C)** Immunofluorescence staining of tumor samples after treatment. Twenty-four hours after the fourth antibody injection, the animals were sacrificed and frozen tumor sections were prepared and detected with anti-human CD3 antibody (red). DAPI was used for DNA staining. **(D)** Quantification of T-cell infiltration based on fluorescence intensity. The results are shown as means ± SDs (n=15). Statistical significance was calculated using the Newman Keuls multiple comparison test (C, E, F, H and I): *P < 0.05, **P < 0.01, and ***P < 0.001; ns, not significant (≥0.05).

A mouse model was further designed to simultaneously compare the efficacy of bsAbs and tsAb against Her2- or VEGFR2-expressing tumors to evaluate the therapeutic index of the tsAb in avoiding antigen evasion. As shown in Figure 5A, MDA-MB-468/Her2 and MDA-MB-468/VEGFR2 tumor cells were injected s.c. into opposite flanks of the same NCG mice, and T cells were administered once the tumor volume had reached approximately 200 mm^3^. The mice were i.v. injected with Her2/CD3 HCT, VEGFR2/CD3 LG, their combination, or Her2/VEGFR2/CD3 (SO), and tumor size assessments were conducted. As shown in Figure 5B, mice treated with either bsAb alone exhibited nearly complete regression of the target-specific tumor xenograft. In addition, progressive growth of either Her2- or VEGFR2- expressing tumors on the opposite flank was also observed in mice treated with the bsAb specific for only VEGFR2 or Her2. In contrast, almost complete regression of both genetic MDA-MB-468 tumors was observed in mice treated with the bsAb combination or tsAb, indicating that tsAb potentially circumvented the immune evasion of tumors undergoing monospecific targeted drug treatment. Immunofluorescent staining of the tumors indicated increased infiltration of CD3+ T cells in either Her2- or VEGFR2-expressing tumors treated with the corresponding bsAb and an increase in the overall T-cell distribution in both tumors treated with the bsAb combination or tsAb (Figure 5C). Evidently, the statistical analysis confirmed that tsAb induced insignificant T-cell infiltration into either antigen-expressing tumor compared with the activity obtained with the bsAb combination (Figure 5D), suggesting that the reformulation of bsAb into the tsAb format resulted in efficient T-cell redirection activity toward tumors expressing either target. Taken together, these results suggest that tsAbs with increasing antigen target specificity exhibit increased reactivity toward tumors with heterogeneous antigen expression or undergoing immune evasion, which leads to systemic tumor toxicity and an overall improved therapeutic index.

## Discussion

The development of multifunctional antibodies has markedly affected the field of cancer therapy, resulting in the development of numerous molecular design approaches to engineering molecules with improved preclinical and clinical performance 3, 4, 31. Detailed investigations into rational molecular designs to optimize IS formation between target cells and effector cells and achieve superior antitumor activity have not generally been addressed in this area 23, 25, 32. To the best of our knowledge, this study represents the first comprehensive preclinical examination of the effects of construct design on the *in vitro* and *in vivo* behaviors of T-cell-redirecting tsAbs. Most importantly, the current study clearly describes the flexibility of a site-specific recombination strategy to simply optimize the cancer cell/T-cell interaction and stimulation triggered by tsAbs.

Building on the understanding of cellular immunology, specifically the antigen recognition of malignant cells, the molecular basis of T-cell activation has now been observed with immune checkpoint-targeted monoclonal antibodies and CAR-T cells, as well as T-cell engagers 33, 34. As an example of effective immunotherapy, the dual specificity of HER2 BATs targeting Her2/CD3 leads to improved outcomes in the treatment of Her2-low metastatic breast cancer 35. Despite significant advances, a majority of malignancies including more immunogenic tumor types, remain unresponsive to T-cell therapy 36, 37. Here, we explored the potential of multitargeting with different antibodies combined into a single protein that confers enhanced T-cell recognition of Her2- and/or VEGFR2-expressing tumor targets. Considering the major role of VEGFR2 in VEGF-induced angiogenesis in human cancer, the finding of high VEGFR2 expression in Her2-positive breast cancer provides scientific rationale for studying the clinical activity of therapeutic blockade of VEGFR2 in the clinically aggressive breast cancer subtype 30, 38. However, no reports of molecular designs to redirect T cells to cotarget Her2/VEGFR2 in cancer therapy are available. The design of T-cell engagers with dual TAA targets is desirable for improved avidity that could result in enhanced antitumor breadth and potency. Of note, our Her2/VEGFR2/CD3 tsAb obtained after structural optimization has strong T-cell-redirecting cytotoxic activity *in vitro* and *in vivo* against Her2- and/or VEGFR2-positive cells and overcomes the poor targeted avidity of the corresponding bsAb candidates, particularly tumor cells resistant to the combination of Herceptin and Cyramza, which suggests promising clinical application in patients with a poor prognosis due to tumor relapse or resistance to conventional antibody therapy.

To date, several strategies have been developed to synthesize bsAbs, including recombinant approaches such as diabodies 39, BiTEs 40, DARTs 41, non-CDR loop fusion 28, and IgG-based formats such as Triomab 42, DVD-Ig 43, and two-in-one antibodies 44. Progress in antibody engineering and biorthogonal chemistry has facilitated the preparation of homogeneous site-specific antibody conjugates using methods such as disulfide rebridging 45, SMARTag 46, unnatural sugars 47, noncanonical amino acids 48, or proximity-induced antibody conjugation (pClick) 49. However, these approaches are all designed to include another antibody specific to the site of the primary antibody, and the molecular structures are generally settled. Because the IS between an antigen-presenting cell and a T cell is essential for T-cell activation, the connection flexibility of two or three antibodies determines the geometries of different formats of these molecules and has a major impact on efficacy 23, 24. To design a unique format with optimal T-cell-redirecting activity toward either Her2 or VEGFR2 on cancer cells, we modified the recombination approach using various linker designs for the site-specific fusion of Her2 (2Rs15)- or VEGFR2 (3VGR19)- specific nanobodies to various sites of the CD3 Fab (SP34). We selected a rigid α-helical PD linker (the linker between the lipoyl and E3 binding domains in pyruvate dehydrogenase) 50 or HE linker 51 to display the nanobody in the N-terminus of Fab to reduce interference between each domain, and flexible (G_4_S)_3_ linker to the C-terminus of the Fab connecting nanobody to produce the active form of nanobody domain as in previous studies 52. In addition, to spatially separate the nanobody inserts and Fab backbone, an antiparallel coiled-coil stalk was used as a rigid linker to connect the nanobody and CD3 Fab to increase the stability of the structures. A similar linker strategy was previously used successfully to fuse EPO, GCSF and Exendin-4 into CDR or non-CDR loops of human or bovine antibodies 28, 53-56. Different from TriTACs 57 and TriTE 20, which showed impaired functionalities due to steric hindrance, our Fab-based bsAbs and tsAbs using the special linker design and the site-specific recombination strategy potentially retained the affinity of each antibody domain. This recognized method for bsAb or tsAb design produces a variety of structural options and thus results in the different scenarios in which an artificial IS forms when mimicking physiological membrane spanning or does not efficiently form when distal or close epitope binding is applied. Here, 2Rs15 fused to the C-terminus of the SP34 HC and 3VGR19 grafted to LS171-LD173 of the Fab generated the best IS formation between target cells and T cells and therefore induced highly efficient tumor growth eradication of Her2- and/or VEGFR2-positive cancers. A potential reason for the different fusing positions of 2Rs15 and 3VGR19 to SP34 could be inferred from the recognized epitope location at the membrane region located at either the membrane proximal or distal region of the Her2 or VEGFR2 antigen. Thus, access of the 2Rs15 to SP34 C-terminus or 3VGR19 to S171-D173 of SP34 LC could likely provide the necessary geometry to enable optimal target and T-cell interactions. It is worth noting the requirement of personalized optimization of the tsAb design if targeting other TAAs, as another work suggested the optimal structure of CD19/CD22/CD3 tsAb was obtained by fusing both CD19- and CD22-specific antibodies to the C-terminus of the HC and LC of SP34 Fab 58. Collectively, this study underscores the importance of optimizing the IS geometry, which directly affects tsAb activity.

The initial goal of this study was to create an optimal tsAb construct targeting Her2 and VEGFR2 double- or single-positive cancer cells without immune evasion, which would allow long-term, repeated administration with significant efficacy and safety. Therefore, we report the construction of two TAA-specific nanobodies on the Fab-based backbone and characterize these constructs in a number of cancer cells. Accordingly, IgG1-format bsAbs cause nonspecific T-cell activation via an Fc interaction with accessory cells and trigger the release of inflammatory cytokines from T cells (cytokine storm), and nearly all patients thus develop symptoms of CRS 29, 59, 60. Moreover, the reduced nonspecific activity of IgG4 potentially improves the safety of IgG-like bsAbs, and the recent CD38/CD3/CD28 tsAb developed by Sanofi efficiently induces myeloma growth suppression and T-cell stimulation 19; however, we did not expect timely cessation of the potential toxic activity due to the prolonged half-life. Unlike full antibodies, Fab-based proteins reveal distribution and elimination half-lives on the order of hours 61, 62, and these short half-lives enable precise control of the level of Fab-based T-cell engagers within patients, which enhances their potential safety. In addition, the challenge of short protein half-life has been met by the use of constant infusion pumps such as in the clinical use of blinatumomab 63. In this study, Her2/VEGFR2/CD3 (SO) with an every-other-day treatment schedule demonstrated impressive *in vivo* tumor growth inhibition but no observed adverse effects, suggesting the potential combination of improved efficacy and safety intervention.

The presented animal study highlights an additional point regarding the efficacy differences between tsAb and bsAb combination. While Her2/VEGFR2/CD3 (SO) showed significant improvement of tumor growth inhibition and T-cell infiltration over the coadministration of bsAbs, the enhancement was not observed in immune evasion models. Further comprehensive *in vivo* comparison studies, including dose titration analysis against tumors with various expression levels of single or dual antigens, are necessary to determine the treatment advantage of tsAb over bsAb combination.

## Conclusions

In conclusion, we describe the development of a precisely designed tsAb via a modified site-specific recombination strategy for T-cell redirection to treat Her2- and/or VEGFR2-expressing cancers. This strategy imparted apparent advantages such as well-defined, site-specific recombination and, in this case, maneuverability of IS formation between target and effector cells. A tunable homogeneous system allows unification of the activity and kinetics of engineered antibodies. Importantly, the structurally optimized tsAb exhibited overall superior *in vitro* and *in vivo* T-cell-retargeting activity and tumoricidal properties compared with the corresponding optimal bsAbs, their combination or other formats of tsAbs. Therefore, our site-specific recombination strategy desirable to generate tsAbs with optimal structure is expected to simplify the development of tsAbs for the application of immune-cell-based therapy to target other types of cancers as well as nononcology indications.

## Materials and Methods

### Cell lines and antibodies used

Human breast cancer cell lines (MDA-MB-468, MCF-7 and MDA-MB-435), a prostate cancer cell line (PC-3), and a pancreatic beta cell line (ASPC-1) were purchased from the ATCC, and a virus-producing cell line (HEK293FT) was purchased from Thermo Fisher Scientific. Her2- and/or VEGFR2- positive MDA-MB-468 cell lines were generated by lentivirus transduction. All these cells were maintained in DMEM high-glucose medium (HyClone) supplemented with 10% heat-inactivated fetal bovine serum (Genstar Technologies Co Inc). The human T-cell leukemia cell line (Jurkat), human PBMCs and T cells were cultured in RPMI 1640 medium (HyClone) with 10% heat-inactivated fetal bovine serum (Genstar Technologies Co Inc). All of the abovementioned media contained 2 mM L-glutamine, MEM nonessential amino acids, 1 mM sodium pyruvate, 100 units/mL of penicillin and 100 μg/mL streptomycin. Unless otherwise specified, all media and supplements were purchased from HyClone. The HEK293 suspension cell line was obtained from Sino Biological Inc. and cultured in 293TII medium (Sino Biological Inc.). All cells were authenticated by short tandem repeat (STR) and cultured within 15 passages from thawing.

All the antibodies used in western blotting, flow cytometry, ELISA and immunofluorescent staining are listed in Table S2.

### Construction of the Her2/CD3 HC and VEGFR2/CD3 LC fusion

The genes encoding the HC and LC of anti-CD3 Fab (clone: SP34) were synthesized by GenScript and cloned into the pGAGGS vector supplied by Prof. Guochai Zhong (Shenzhen Bay Laboratory). Anti-Her2 nanobody (clone: 2Rs15d) or VEGFR2 specific nanobody (clone: 3VGR19) were synthesized by GenScript and further inserted into the specific sites of the HC or LC of SP34 by various linker designs. The rigid peptide linker from pyruvate dehydrogenase (PD linker) and the α-helical EAAAK linker (HE linker) were used to expand the nanobody to the N-terminus of the HC or LC of the SP34 Fab. The flexible GGGGS linker was selected to fuse the nanobody to the C-terminus of either the HC or LC of the Fab. For the site-specific grafting strategy, we applied coiled-coil stalk peptide linkers to either the end of HS184 and HL187 by replacing HS185-HG186, or the end of LS171 and LD173 by replacing LK172 of SP34. The linker sequences are listed in Table S1. The genes encoding all the HC or LC fusions were obtained by overlap extension PCR, further framed to the pCAGGS vector and confirmed by DNA sequencing.

### Expression and purification of antibody fusions

The fusion proteins were expressed through transient transfection of HEK293 suspension cells with the cloned expression vectors, according to the manufacturer's protocol. Briefly, 50 mL of a HEK293 cell suspension containing 5 × 10^7^ cells was seeded in a 125-mL shaking flask. Defined amounts of plasmids encoding HC and LC were diluted in 1 mL Opti-MEM medium and added to 1 mL Opti-MEM containing 60 μL of 1 mg/mL PEX MAX (Polysciences, Inc.). After the plasmids were incubated with PEI MAX for 30 min at room temperature, the mixture was added to the cell suspension. The cells were then shaken at 125 rpm in a 5% CO_2_ environment at 37 °C. Culture medium containing the secreted fusion proteins was harvested 72 h after transfection. The fusion proteins were purified by Protein G and size-exclusion chromatography. The purified protein was analyzed by SDS-PAGE.

### Analysis of binding activity by ELISA and flow cytometry

The apparent binding affinity was evaluated by ELISA: 96-well ELISA plates were coated with Her2, VEGFR2, or CD3 extracellular domain (ECD) overnight at 4 °C and then blocked with 2% BSA in PBS for 1 h at 37 °C. A series of diluted antibody fusions were then added, and the plates were incubated for 2 h at room temperature. HRP-labeled polyclonal anti-human kappa light chain (1: 2000) was then added, and the plates were incubated for 2 h at room temperature. After washing, blue color development was performed using a 3,3,5,5-tetramethylbenzidine (TMB) substrate and quantified using a Cytation 5 Cell Imaging Multi-Mode Reader (Agilent) with excitation at 450 nm. The data were plotted and analyzed using GraphPad Prism by nonlinear regression with the log (agonist) vs. response model.

The binding activity of antibody fusions to human CD3 was determined by flow cytometry using human T lymphocytes. Jurkat cells were incubated with primary fusion antibodies (100 nM) for 1 h at 4 °C. After several washes, the cells were incubated with APC-conjugated anti-human kappa antibody and then used for data acquisition with an Attune NxT flow cytometer (Thermo Fisher Scientific). All the flow cytometry data were analyzed with FlowJo 10.0.6 (Treestar).

### Microscopy analysis of immunological synapse formation

Immunofluorescence-based conjugation studies were performed based on Her2- or VEGFR2- transduced MDA-MB-468 cells with Jurkat cells. The target cells (MDA-MB-468/Her2-mCherry or MDA-MB-468/VEGFR2-GFP) were seeded onto poly-L-lysine coated coverslips, and incubated overnight at 37 °C with 5% CO2. After washing, the target cells were further incubated at 37 °C with Jurkat cells in the presence of various bsAb formats at a concentration of 10 nM for 2 h. The wells were then gently washed with PBS, fixed with 3% paraformaldehyde, permeabilized with 0.2% Triton X-100, and further stained with mouse anti-LFA-1 antibody and Alexa Fluor 488- or Alexa Fluor 555-conjugated anti-mouse IgG. Images were acquired on a laser-scanning confocal microscope (Nikon A1R confocal microscope) under an Oil Immersion Objective (100×). Processing and analysis were performed using FluoView software (FV10-ASW version 01.07, Olympus).

### *In vitro* cytotoxicity assay

Cytotoxicity assays were performed using human PBMCs or activated T cells as the effector cells, and cancer cells as the target cells. PBMCs were isolated from healthy donors by Ficoll density gradient centrifugation using standard procedures and incubated in tissue culture flasks for >1 h to remove adherent cells. To obtain activated T cells, human PBMCs were activated with plate-bound anti-CD3 antibody (clone OKT3) and 2 μg/mL soluble anti-CD28 antibody (clone CD28.2) and maintained in RPMI-1640 media supplemented with 10% FBS and 100 IU/mL recombinant human IL-2 (GenScript). For the cytotoxicity study, the target cells (1×10^4^ cells) were mixed with PBMCs or T cells (1×10^5^ cells) and incubated with different concentrations of the bsAbs or tsAbs for 24 h at 37 °C in 5% CO_2_. Cytotoxic activity was determined by measuring the lactate dehydrogenase (LDH) levels in the cultured supernatant using the Cytotox-96 nonradioactive cytotoxicity assay kit (Promega). The percent cytotoxicity was calculated as follows: % cytotoxicity = [(absorbance experimental - absorbance spontaneous average)/(absorbance maximum killing average - absorbance spontaneous average)] ×100.

### T-cell activation analysis

Expanded T cells were incubated with bsAb- or tsAb-bound target cells for 24 h at 37 °C. T-cell activation was analyzed by flow cytometry using PE-conjugated anti-human CD8, FITC-conjugated anti-human CD25, and APC-conjugated anti-human VEGFR2/KDR antibodies. Release of IL-2, IFN-γ and TNF-α in the cultured supernatant was measured with an enzyme-linked immunosorbent assay (ELISA) kit (Thermo Fisher Scientific). The results are shown as means from duplicated samples ± SDs.

### Pharmacokinetic study

A pharmacokinetic study of the bsAbs (Her2/CD3 HCT and VEGFR2/CD3 LG) and TsAb Her2/VEGFR2/CD3 (SO) was performed in female BALB/c mice. The animals were randomized into different treatment groups and were intravenously administered a single dose of 1 mg/kg. Serum samples were obtained at various times over 48 h after injection for bioanalytical measurement. The levels of antibodies in mouse serum were quantified by sandwich ELISA as described previously. In brief, the antibodies were captured with human Her2-Fc or VEGFR2-Fc, and detected with goat anti-human kappa biotinylated polyclonal antibody. The dynamic range for the assay was 0.5 ng/mL to 500 ng/mL. The circulation half-life was calculated by fitting the data to a one-phase exponential decay equation for the bsAbs and tsAb (GraphPad Prism, version 5.04).

### *In vivo* efficacy study in the PC-3 tumor model

All efficacy studies were conducted with 6- to 8-week-old male NOD-Prkdcem26Cd52Il2rgem26Cd22/NjuCrl (NCG) mice. The human prostate cancer cell line PC3 was used to evaluate the efficacy of the Her2/VEGFR2/CD3 (SO) tsAb in comparison to that of the other antibody candidates. For the bsAb comparison study, 2×10^6^ PC3 cells in 50% Matrigel (BD Bioscience) were subcutaneously implanted into the right flanks of mice. Nine days after tumor implantation, the mice received 2.5×10^7^ expanded T cells via intraperitoneal injection, and were then intravenously administrated one of the bsAbs (Her2/CD3 HCT or VEGFR2/CD3 LG), the bsAb combination (1: 1 ratio) or the tsAb at an equivalent dose of 15 nmol/kg or saline every other day. In this study, the mice received additional injections of activated T cells (2.5×10^7^ cells) on day 15 to supplement the effector cells.

To investigate the activity against tumors resistant to conventional antibodies, the mixture of 2×10^6^ PC3 parental or resistant cells with 2×10^6^ human PBMCs in 50% Matrigel was subcutaneously implanted into the right flanks of mice. On day 7, when the tumor volume had reached 100mm^3^, the mice were injected intraperitoneally with 2×10^7^ expanded human T cells and were then intravenously administrated four doses of Herceptin, Cyramza, or the antibody combination every three days, or seven doses of Her2/VEGFR2/CD3 (SO) or saline at 15 nmol/kg every other day. The tumors were measured twice weekly using calipers. The tumor volume was calculated as the width × length × height. All procedures were approved by the Peking University Shenzhen Graduate School Animal Care and Use Committee with Approval Number AP20210914-01 and performed according to national and international guidelines for the humane treatment of animals.

### *In vivo* efficacy in the dual-tumor model

NCG mice were inoculated with 5×10^6^ MDA-MB-468/Her2 cells suspended in 0.2 mL DPBS/Matrigel (1: 1, v/v) in the left flank on day 0. On day 7, the same mice were given 10×10^6^ MDA-MB-468/VEGFR2 cells suspended in 0.2 mL of DPBS/Matrigel (1:1, v/v) in the right flank. Once the tumor volume of each group reached 100 mm^3^, the mice were treated intraperitoneally with 3×10^7^ expanded human T cells and then administered an intravenous injection of one of the bsAbs (Her2/CD3 HCT or VEGFR2/CD3 LG), the bsAb combination (1: 1 ratio) or tsAb at an equivalent dose of 15 nmol/kg or saline every other day. These dual tumor sizes were measured and calculated.

### Cytokine release assays

Fifty microliters of murine blood was drawn 24 h after bsAbs or tsAb infusion to determine the serum IL-2, IFN-γ, and TNF-α cytokine concentrations using the above-described ELISA kits.

### Immunofluorescence staining of tumor tissue

To determine whether tumor growth inhibition was correlated with T-cell-redirecting activity, we analyzed the changes in infiltrating lymphocytes and vasculature in the tumor area after treatment. NCG mice were injected subcutaneously with PC3 cells (2×10^6^ cells/mouse). Once the tumors reached 100 mm^3^, the animals were intraperitoneally injected with 2.5×10^7^ expanded T cells and then given an intravenous injection of one of the bsAbs (Her2/CD3 HCT or VEGFR2/CD3 LG), the bsAb combination (1: 1 ratio) or tsAb at an equivalent dose of 15 nmol/kg or saline every other day. After the additional injection of activated T cells, the mice were scrificed, and tumor samples were collected and frozen for sectioning and mounting on slides. The slides were incubated with anti-human CD3 antibody (FITC-conjugated secondary antibody), and were further subjected to nuclear counterstaining with DAPI. Immunofluorescence observation was performed under a Cytation 5 Cell Imaging Multi-Mode Reader (BioTek). Quantitative analysis of the tissue slides was performed using ImageJ software. Statistical results were obtained from two tumor slides, and four regions from each slide were viewed.

### Immunohistochemical study

After the treatment of PC3 xenografted mice, tumor samples were collected and fixed in 4% formaldehyde and embedded in paraffin. Immunohistochemistry was performed with antibodies against human Her2 (Sangon Biotech, D163227), VEGFR2 (BOSTER, A00901-2), or granzyme B (ab134933, Abcam) to detect a functional marker for activated T cells. Antibody binding was revealed by the addition of 3.30-diaminobenzidine substrate. Tumor tissues were further counterstained with hematoxylin. Immunohistochemistry staining was examined using a Cytation 5 Cell Imaging Multi-Mode Reader (BioTek).

### TUNEL and Ki67 staining

Twenty-four hours after the final injection of antibodies, the mice were sacrificed and tumor samples were frozen immediately in preparation for sectioning and mouting on slides. TUNEL staining was performed as follows: frozen sections were subjected to two 10-min routine rinses with PBS, treated with 0.1% Triton X-100 in PBS for 2 min on ice, rinsed again in PBS and incubated at 37 °C with a terminal deoxynucleotidyl transferase-mediated dUTP nick end labeling (TUNEL) reaction mixture for 60 min. Ki67 staining was conducted by rinsing the sections with PBS. The sections were incubated overnight with a monoclonal rabbit anti-Ki67 antibody as the primary antibody, washed with PBS, and then incubated with PE-conjugated anti-rabbit IgG as the secondary antibody at 37 °C for 50 min. After nuclear counterstaining with DAPI, the slides were analyzed under a Cytation 5 Cell Imaging Multi-Mode Reader (BioTek). Quantitative analysis of the tissue slides was performed using ImageJ software. Statistical results were obtained from two tumor slides, and four regions were viewed from each slide.

### Histological examination

PC3 parental and resistant xenografted mice were treated with saline, Herceptin, Cyramza, the combination of Herceptin and Cyramza, or Her2/VEGFR2/CD3 (SO). For the histological examination of toxicity, the mice were anesthetized after treatment, and mouse organ tissues including the heart, kidney, liver, lung and spleen, were collected and perfused with PBS. After resection, the paraffin-embedded sections were stained with hematoxylin-eosin and observed under a Cytation 5 Cell Imaging Multi-Mode Reader (BioTek).

### Statistical analysis

Statistical analyses were conducted with GraphPad Prism version 8.4.3 software (GraphPad Software, LLC). Data are presented as the mean± SD, and significance was determined using the Newman Keuls multiple comparison test, unless otherwise noted.

## Supplementary Material

Supplementary figures.

## Abbreviations

bsAbs: bispecific antibodies
HCT: heavy chain C-terminal fusion
HE: α-helical EAAAK linker
HG: heavy chain grafting fusion
HNT: heavy chain N-terminal fusion
IS: immunological synapse
LCT: light chain C-terminal fusion
LG: light chain grafting fusion
LNT: light chain N-terminal fusion
PBMCs: peripheral blood mononuclear cells
PD linker: pyruvate dehydrogenase linker
SO: structural optimization
tsAbs: trispecific antibodies
TUNEL: terminal deoxynucleotidyl transferase dUTP nick end labelling
