# Supplementary material for: A Novel Her2/VEGFR2/CD3 trispecific antibody with an optimal structural design showed improved T-cell-redirecting antitumor efficacy
Source: Theranostics. 2022 Nov 14;12(18):7788–803. doi: 10.7150/thno.75037 (PMC9706591; doi:10.7150/thno.75037)
Supplement: Supplementary file 1 — Supplementary figures. [file thnov12p7788s1.pdf]

A Novel Her2/VEGFR2/CD3 trispecific antibody with an optimal structural design showed improved T-cell-redirecting antitumor efficacy

Dong Liu[1,§], Xuexiu Qi[1,§], Xiaoyi Wei[1], Lijun Zhao[1], Xuechun Wang[1], Shuhong Li[1], Zhidong Wang[1], Licai Shi[1], Jie-an Xu[1], Mei Hong[1], Zhong Liu[2], Lili Zhao[3,*], Xiankun Wang[1,4], Bo Zhang[1,4], Yuhan Zhang[5], Feng Wang[5], Yu J. Cao[1,6,*]

[1] State Key Laboratory of Chemical Oncogenomics, Guangdong Provincial Key Laboratory of Chemical Genomics, Peking University Shenzhen Graduate School, Shenzhen, Guangdong, 518055, China

[2] Lunan Pharmaceutical Group Co., Ltd, Feixian County, Shandong, 273400, China

[3] National Engineering Laboratory of High Level Expression in Mammalian Cells, Feixian County, Shandong, 273400, China

[4] Institute of Neurological and Psychiatric Disorders, Shenzhen Bay Laboratory, Shenzhen, Guangdong, 518132, China

[5] Key Laboratory of Protein and Peptide Pharmaceuticals, Beijing Translational Center for Biopharmaceuticals Institute of Biophysics, Chinese Academy of Sciences Beijing 100101, China

[6] Institute of Chemical Biology, Shenzhen Bay Laboratory, Shenzhen, 518132, China

§ These authors contributed equally

* To whom correspondence should be addressed: zhaolili1820@126.com; joshuacao@pku.edu.cn

Supplementary Table S1. Linker sequences used in bsAbs and tsAbs

**Linkers**	**Amino acid sequences**
PD Linker	GGSGRGAAPAAAPAKQEAAAPAPAAKAEAPAAAPAAKAGGSG
HE Linker	EAAAKEAAAKEAAAK
Coiled Coil Linker (ascending peptide)	GGSGAKLAALKAKLAALKGGGGS
Coiled Coil Linker (descending peptide)	GGGGSELAALEAELAALEAGGSG
GGGGS Linker	GGGGSGGGGSGGGGS

Supplementary Table S2. Antibodies used in the experiments

Antibodies	Manufacturer	Catalogue
Goat Anti-Human Kappa-HRP	SouthernBiotech	2060-05
FITC anti-human CD25 Antibody (Clone: BC96)	Biolegend	302604
PE anti-human CD8 Antibody (Clone: SK1)	Biolegend	344706
CD11a (LFA-1alpha) Monoclonal Antibody (Clone: R7.1)	ThermoFisher	BMS102
Goat anti-Mouse IgG (H+L) Highly Cross-Adsorbed Secondary Antibody, Alexa Fluor Plus 488	ThermoFisher	A32723
Goat anti-Mouse IgG (H+L) Highly Cross-Adsorbed Secondary Antibody, Alexa Fluor Plus 555	ThermoFisher	A32727
Purified Rat Anti-Mouse CD31 Clone (Clone: MEC 13.3)	BD Pharmingen	553370
Recombinant Anti-CD3 epsilon/CD3e Antibody, Rabbit Monoclonal	SinoBiological	10977-R801
Goat anti-Rabbit IgG (H+L) Highly Cross-Adsorbed Secondary Antibody, Alexa Fluor 488	ThermoFisher	A11034
Alexa Fluor® 594 goat anti-rat IgG (H+L)	ThermoFisher	A11007
FITC Anti-Human CD3 (Clone: HIT3a)	Biolegend	300306
Alexa Fluor® 647 Mouse anti-Ki-67 (Clone B56)	BD Pharmingen	558615
Goat Anti-Human Kappa- Alexa Fluor 647	SouthernBiotech	2060-31
Anti-Her2/ERBB2 Antibody (APC), Mouse Monoclonal (Clone: 9G1D4B10)	SinoBiological	10004-MM03-A
Anti-VEGFR2/KDR Antibody (APC), Mouse Monoclonal (Clone: 06)	SinoBiological	10012-MM06-A
PE anti-human CD340 (erbB2/HER-2) Antibody (Clone: 24D2)	Biolegend	324406
Anti-VEGFR2/KDR Antibody (Clone: P35968)	BOSTER	BM4256
Rabbit anti-Her2/ERBB2 Polyclonal Antibody	Sangon Biotech	D163227
VEGFR2/KDR/FLK1 antibody	BOSTER	A00901-2
GAPDH Polyclonal Antibody	Bioworld Technology	AP0066
Granzyme B Recombinant Rabbit Monoclonal Antibody	Invitrogen	MA5-29315
HRP-Conjugated Goat Anti-Rabbit antibody	Sangon Biotech	D110058-0025

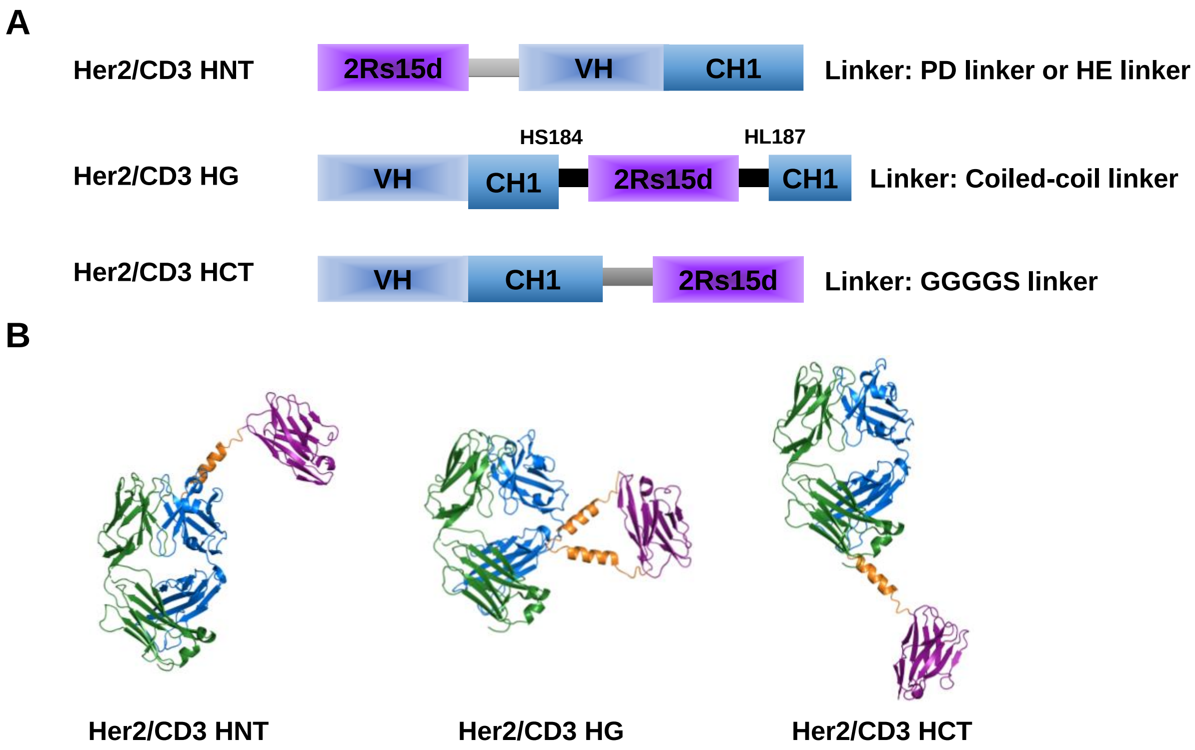

Supplementary Figure S1. The design of different structures of Her2/CD3 bsAbs. (A) Representative sequences of different formats of Her2/CD3 bsAbs: Her2/CD3 HNT, Her2/CD3 HG and Her2/CD3 HCT. (B) Ribbon diagrams depicting the Her2/CD3 bsAbs, where the heavy chain of CD3-specific Fab (SP34) shown in blue was fused with Her2-targeted nanobody (2Rs15) in purple at its N-terminus, S184-L187 and C-terminus.

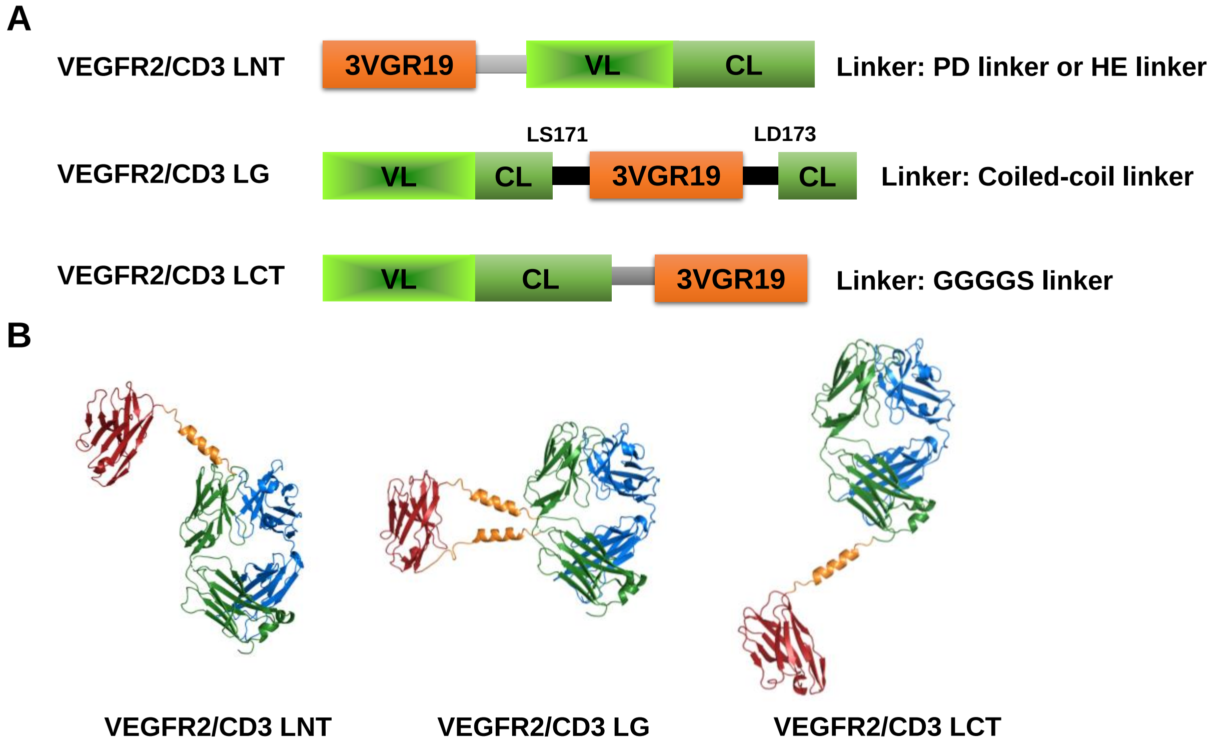

Supplementary Figure S2. The design of different structures of VEGFR2/CD3 bsAbs. (A) Representative sequences of different formats of VEGFR2/CD3 bsAbs: VEGFR2/CD3 LNT, VEGFR2/CD3 LG and VEGFR2/CD3 LCT. (B) Ribbon diagrams depicting the VEGFR2/CD3 bsAbs, where the light chain of CD3-specific Fab (SP34) shown in green was fused with VEGFR2-targeted nanobody (3VGR19) in red at its N-terminus, S171-D173 and C-terminus.

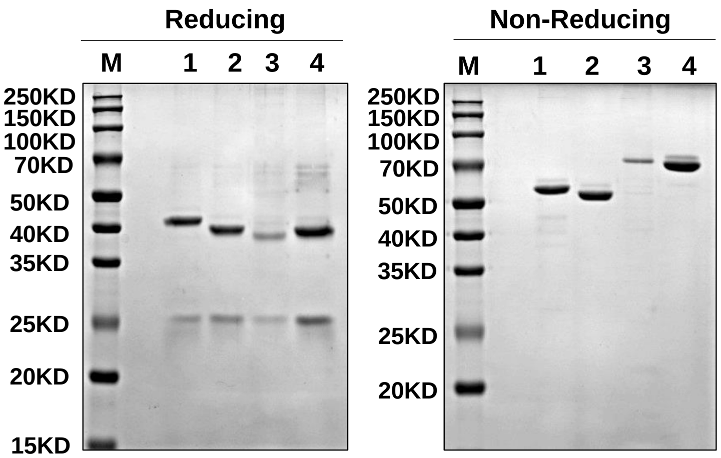

M, Protein ladder
1, Her2/CD3 HNT (PD Linker)
2, Her2/CD3 HNT (HE Linker)
3, Her2/CD3 HG
4, Her2/CD3 HCT

Supplementary Figure S3. SDS-PAGE analysis of different Her2/CD3 bsAbs under reducing and non-reducing conditions.

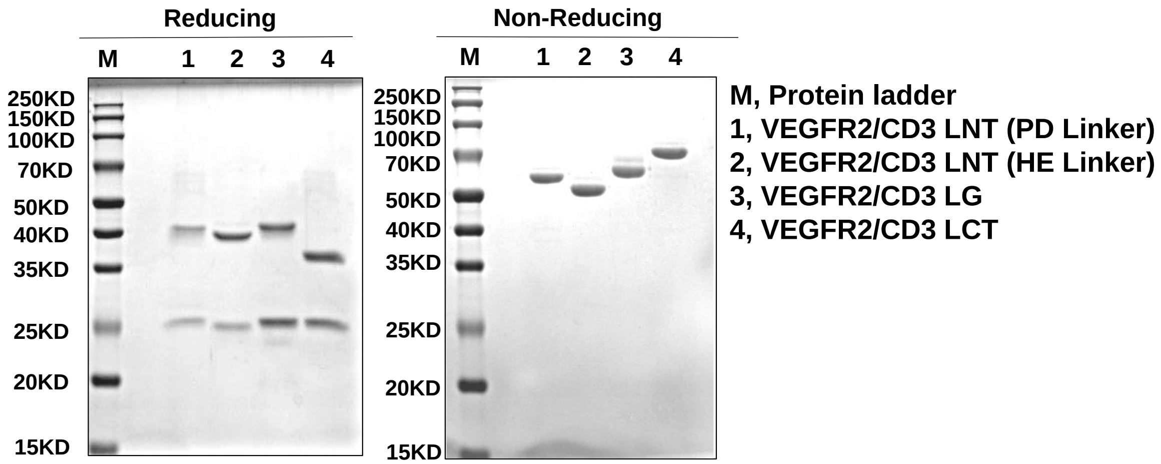

Supplementary Figure S4. SDS-PAGE analysis of different VEGFR2/CD3 bsAbs under reducing and non-reducing conditions.

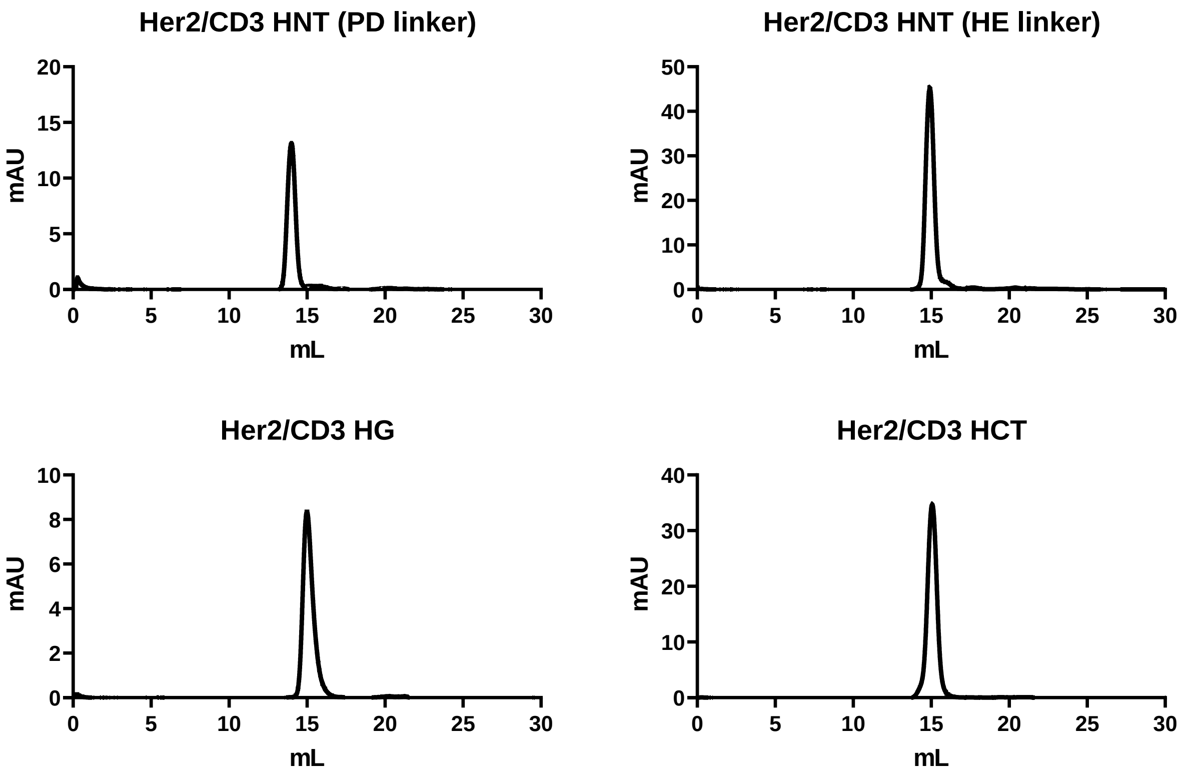

Supplementary Figure S5. Gel-filtration (Superdex 200) profiles of different Her2/CD3 bsAbs.

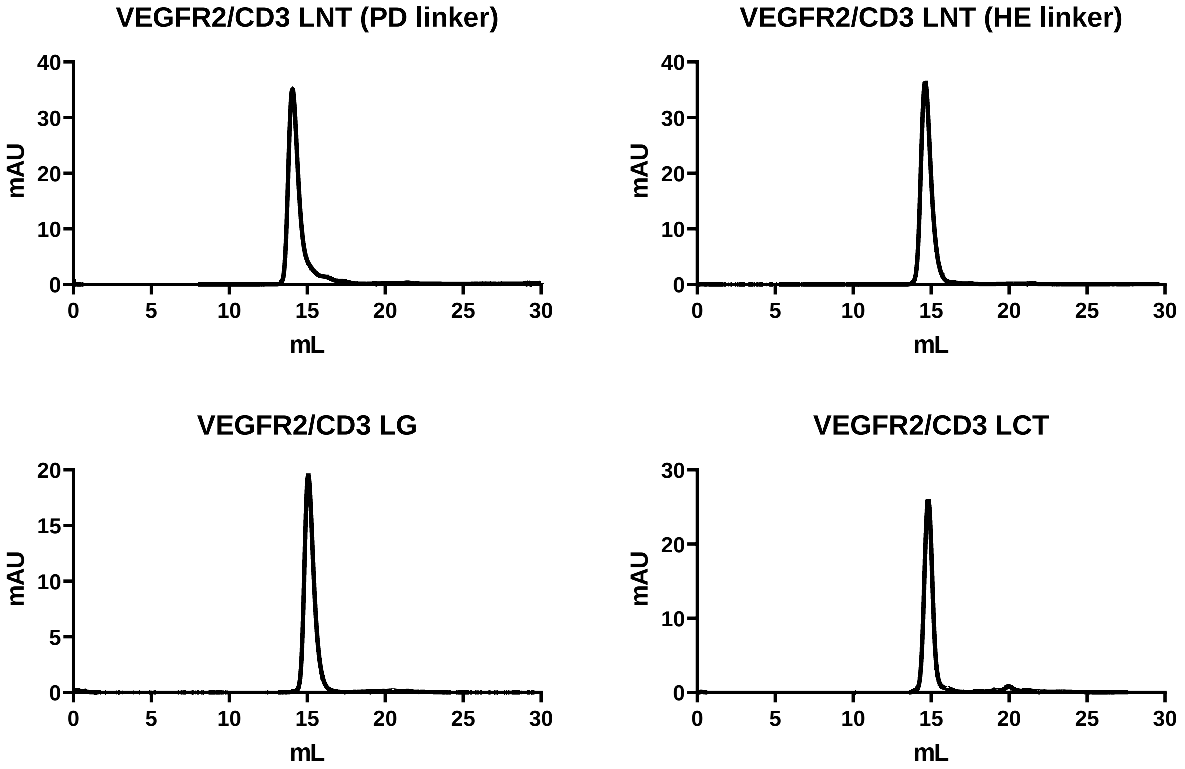

Supplementary Figure S6. Gel-filtration (Superdex 200) profiles of different VEGFR2/CD3 bsAbs.

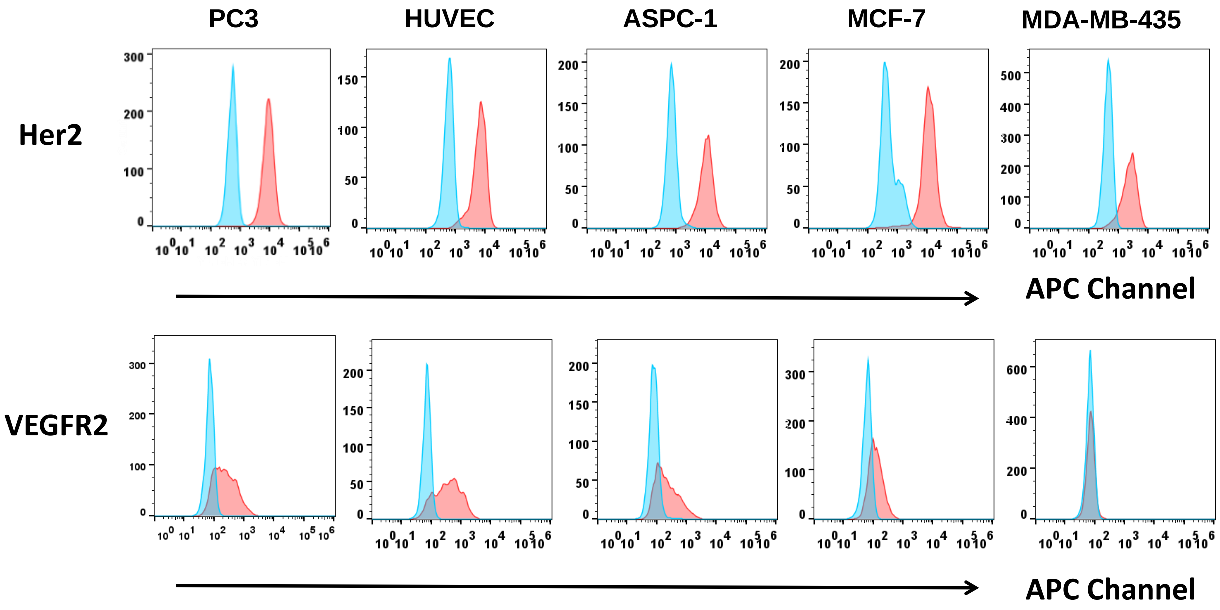

Supplementary Figure S7. Flow cytometry analysis Her2 and VEGFR2 expression on different cell lines. Her2 and VEGFR2 expression was determined with an PE-conjugated anti-Her2 antibody and APC-conjugated anti-VEGFR2 antibody respectively.

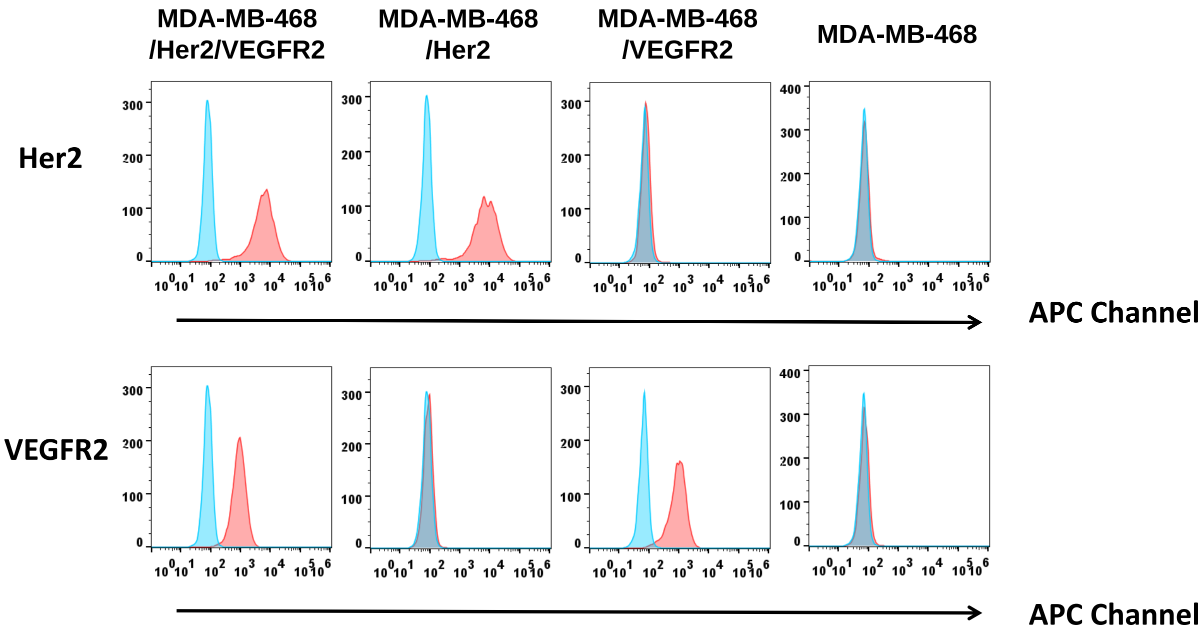

Supplementary Figure S8. Flow cytometry analysis Her2 and VEGFR2 expression on different genetic MDA-MB-468 cancer cells. Her2 and VEGFR2 expression was determined with an PE-conjugated anti-Her2 antibody and APC-conjugated anti-VEGFR2 antibody respectively.

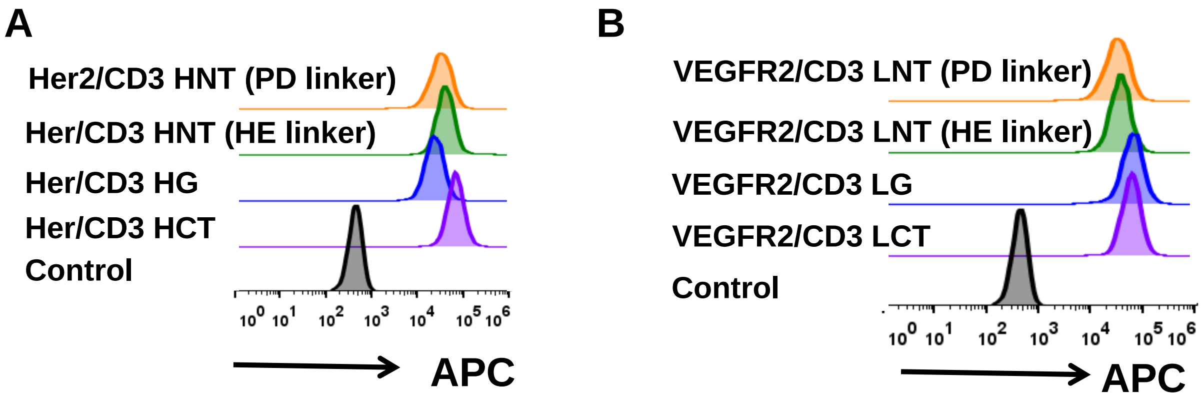

Supplementary Figure S9. Flow cytometry analysis of bsAb constructs binding to CD3 positive Jurkat cells. Cells were consecutively labeled with 100nM Her2/CD3 (A) or VEGFR2/CD3 (B) bsAbs and secondary APC-conjugated anti-human kappa antibody.

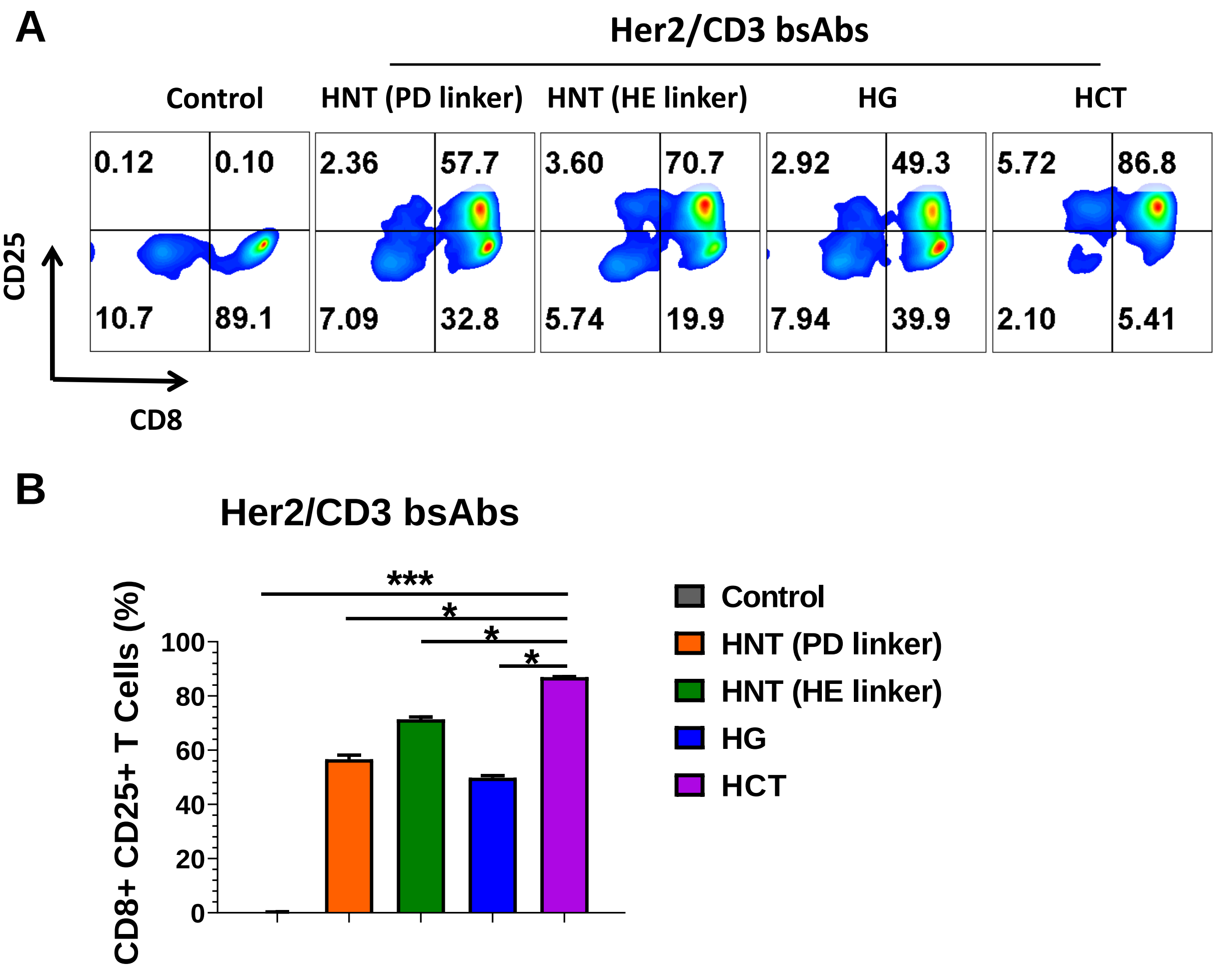

Supplementary Figure S10. Activation of CD8+ T cells in the presence of different Her2/CD3 bsAbs against Her2-expressing MDA-MB-468 cells. (A) T cells were measured by anti-CD8/anti-CD25 antibody cocktails after 24-h incubation with Her2-expressing MDA-MB-468 cells (E: T = 5: 1) in the presence of 10 nM bsAbs. (B) Quantification of CD8+ T cell activation with Her2/CD3 bsAbs by flow cytometry staining for CD25. The data are presented as the means ± SDs, and statistical significance was calculated using Student's t test: *P < 0.05, **P < 0.01, and ***P < 0.001; ns, not significant (≥0.05).

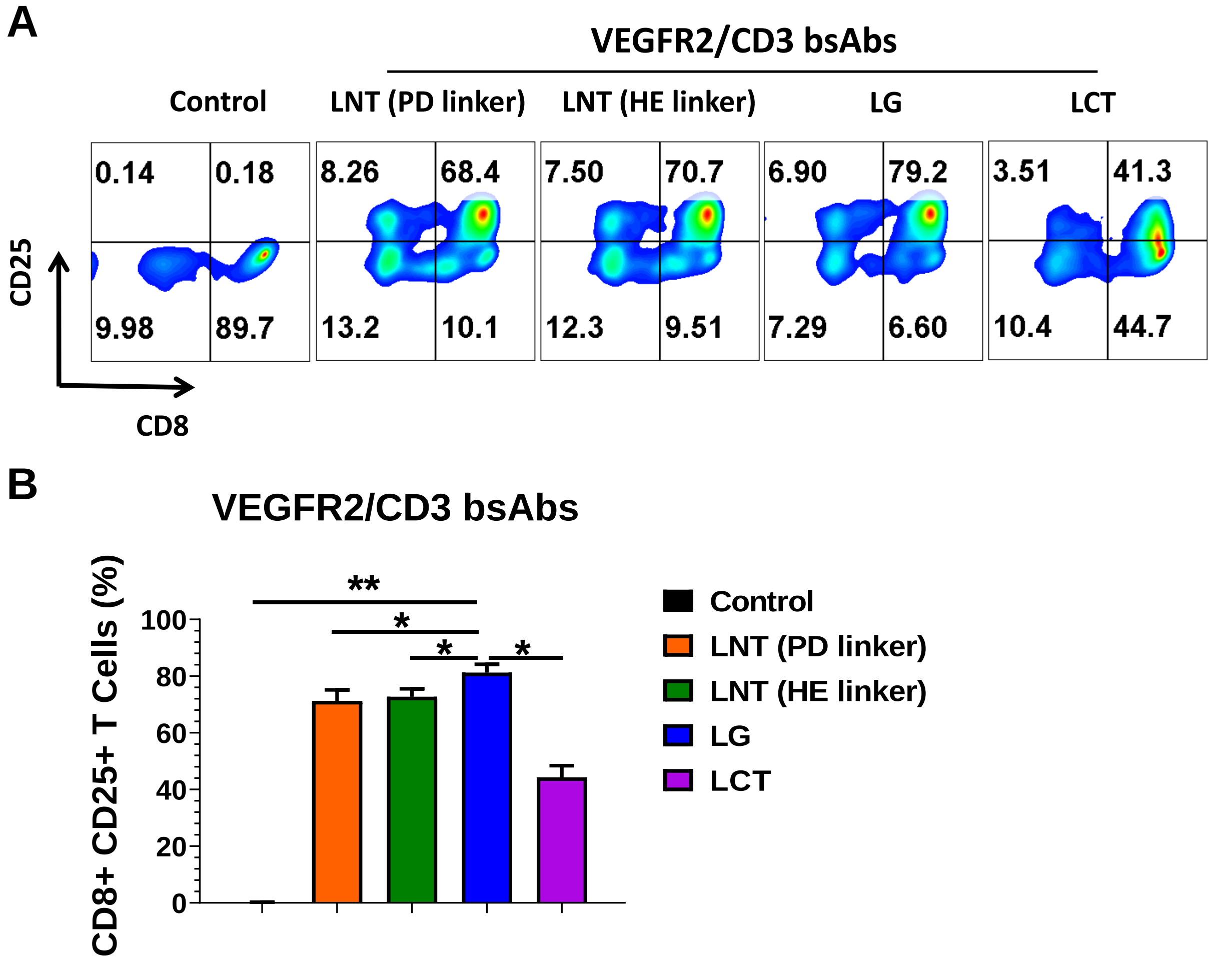

Supplementary Figure S11. Activation of CD8+ T cells in the presence of different VEGFR2/CD3 bsAbs against VEGFR2-expressing MDA-MB-468 cells. (A) T cells were measured by anti-CD8/anti-CD25 antibody cocktails after 24-h incubation with VEGFR2-expressing MDA-MB-468 cells (E: T = 5: 1) in the presence of 10 nM bsAbs. (B) Quantification of CD8+ T cell activation with VEGFR2/CD3 bsAbs by flow cytometry staining for CD25. The data are presented as the means ± SDs, and statistical significance was calculated using Student's t test: *P < 0.05, **P < 0.01, and ***P < 0.001; ns, not significant (≥0.05).

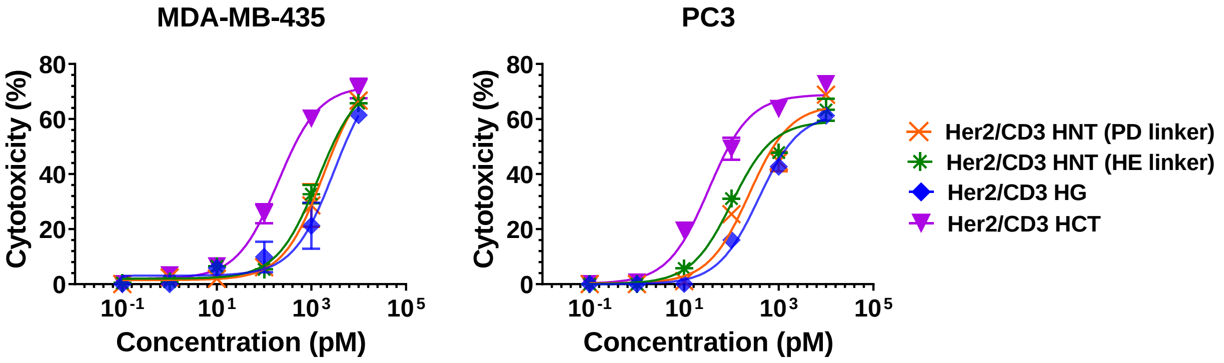

Supplementary Figure S12. Cytotoxicity of T cells against MDA-MB-435 and PC3 cells in presence of different Her2/CD3 bsAbs. The cytotoxicity assays were performed with an E: T ratio of = 10: 1 and after 24 h incubation. The cytotoxicity was assessed by LDH release. The data are presented as the means ± SDs.

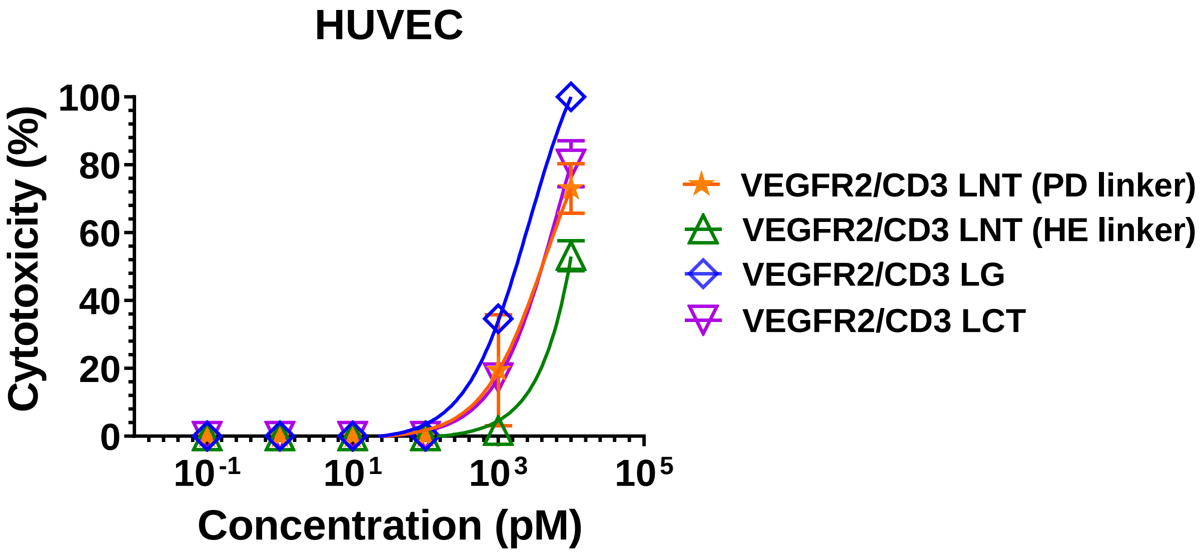

Supplementary Figure S13. Cytotoxicity of T cells against HUVECs in presence of different VEGFR2/CD3 bsAbs. The cytotoxicity assays were performed with an E: T ratio of = 10:1 and after 24 h incubation. The cytotoxicity was assessed by LDH release. The data are presented as the means ± SDs.

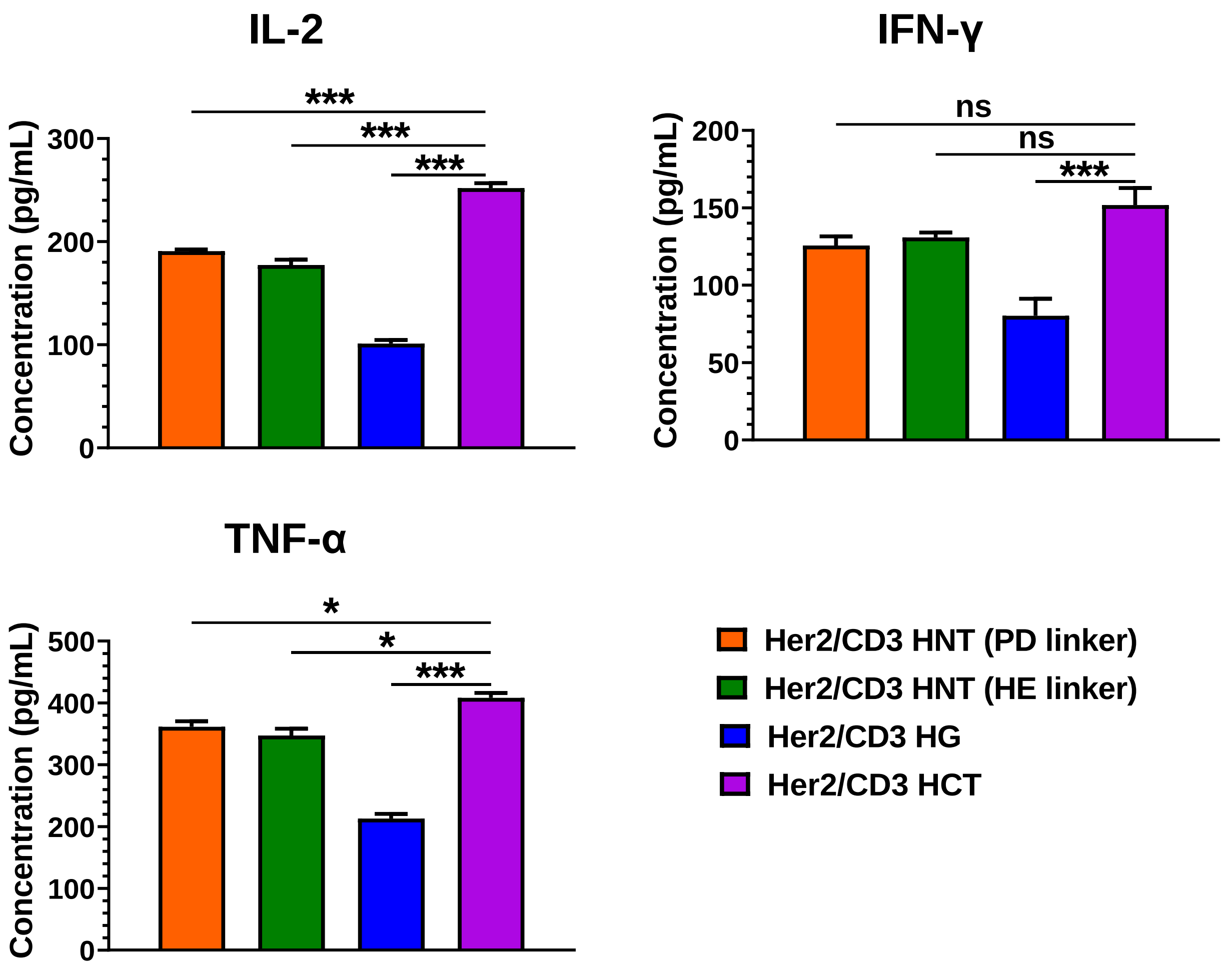

Supplementary Figure S14. Quantification of cytokine levels in the culture of MDA-MB-468/Her2 cells and T cells in presence of different Her2/CD3 bsAbs. IL-2, IFN-γ and TNF-α release was measured by ELISA kit from T cells cultured with MDA-MB-468/Her2 cells (E: T = 5: 1) in the presence of Her2/CD3 bsAbs. The data are presented as the means ± SDs, and statistical significance was calculated using Student's t test: *P < 0.05, **P < 0.01, and ***P < 0.001; ns, not significant (≥0.05).

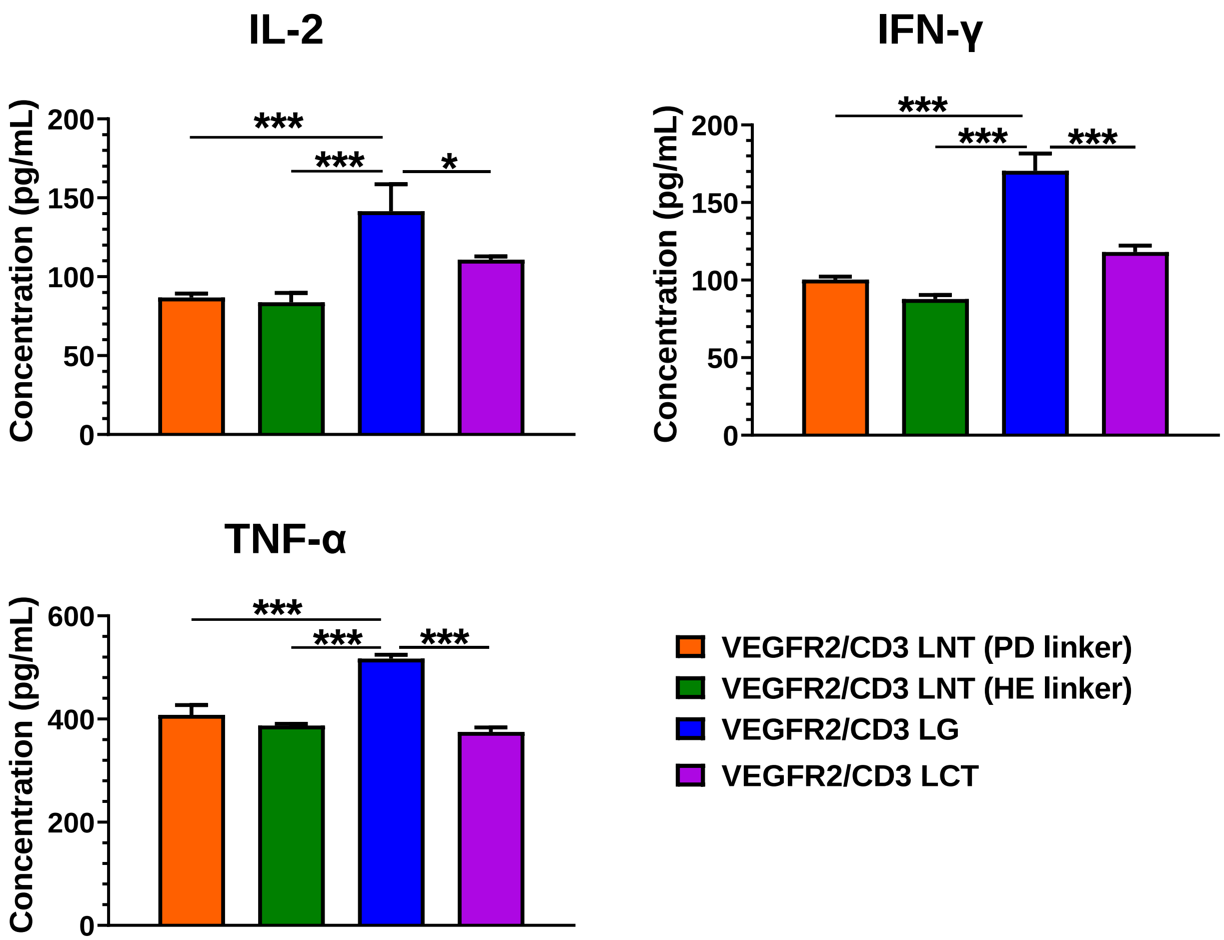

Supplementary Figure S15. Quantification of cytokine levels in the culture of MDA-MB-468/VEGFR2 cells and T cells in presence of different VEGFR2/CD3 bsAbs. IL-2, IFN-γ and TNF-α release was measured by ELISA kit from T cells cultured with MDA-MB-468/VEGFR2 cells (E: T = 5: 1) in the presence of VEGFR2/CD3 bsAbs. The data are presented as the means ± SDs, and statistical significance was calculated using Student's t test: *P < 0.05, **P < 0.01, and ***P < 0.001; ns, not significant (≥0.05).

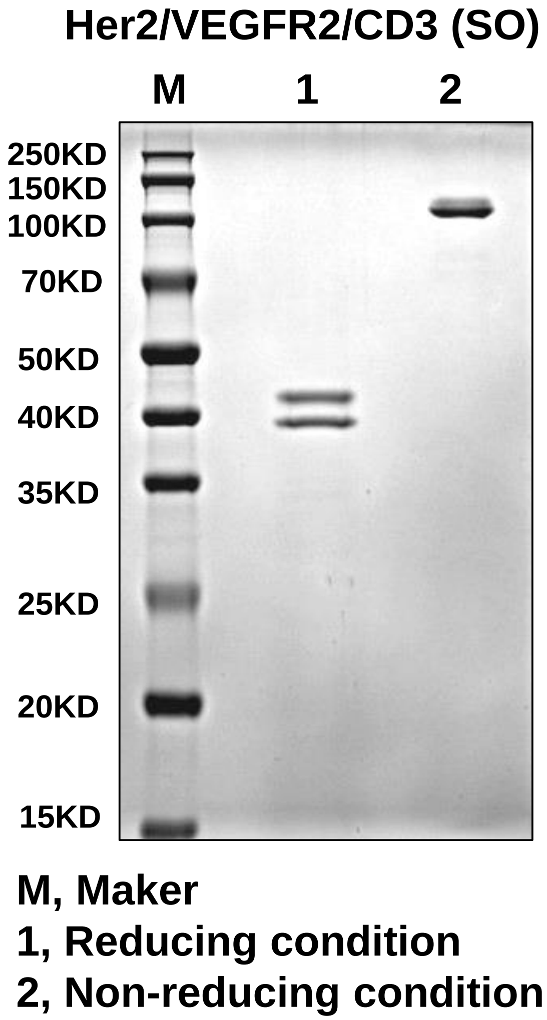

Supplementary Figure S16. SDS-PAGE analysis of the purified tsAbs under nonreducing and reducing conditions.

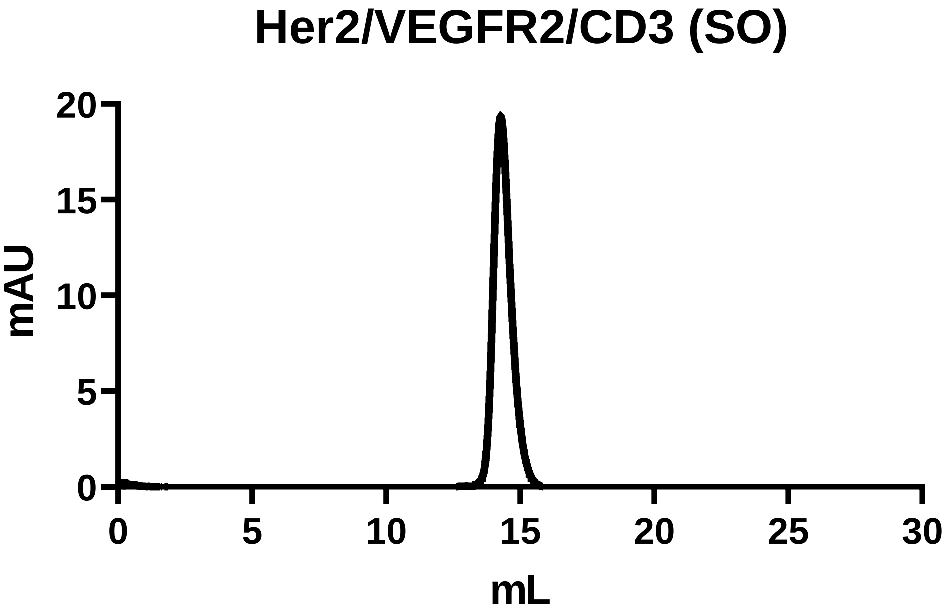

Supplementary Figure S17. Gel-filtration (Superdex 200) profiles of Her2/VEGFR2/CD3 (SO) tsAb.

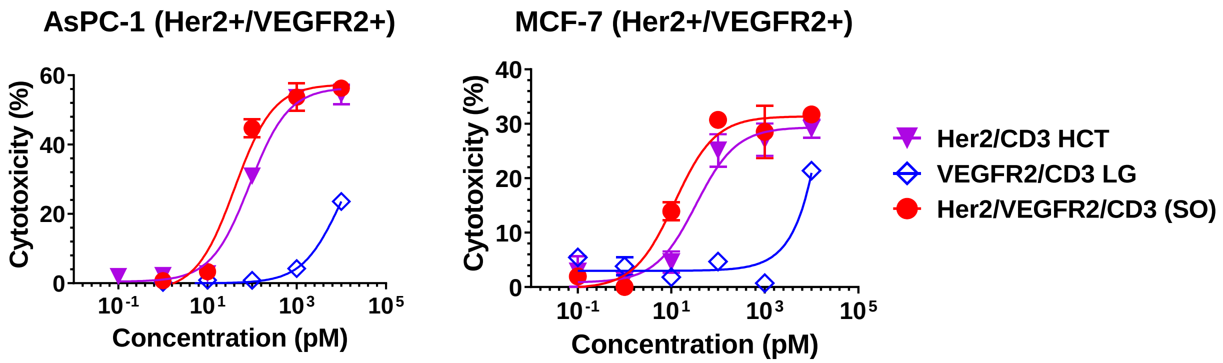

Supplementary Figure S18. Cytotoxicity of different forms of Her2/VEGFR2/CD3 tsAbs redirecting T cells against different Her2/VEGFR2-positive cancer cells. The cytotoxicity assays were performed with an E: T ratio of = 10:1 and after 24 h incubation. The cytotoxicity was assessed by LDH release. The data are presented as the means ± SD.

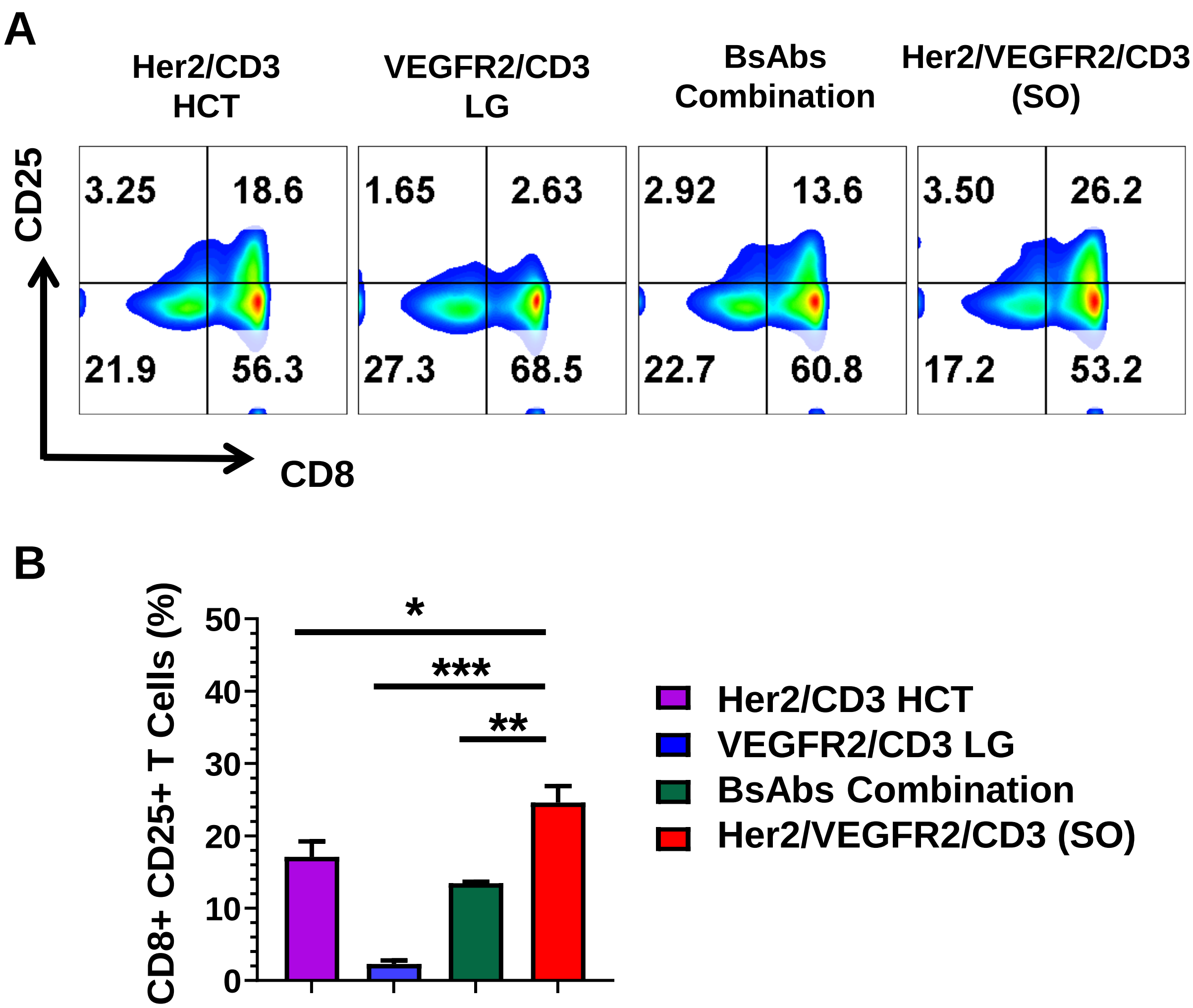

Supplementary Figure S19. Activation of CD8+ T cell activation in the presence of Her2/VEGFR2/CD3 (SO) or the corresponding bsAbs against PC3 cells. (A) T cells were measured by anti-CD8/anti-CD25 antibody cocktails after 24-h incubation with PC3 cells (E: T = 20: 1) in the presence of 1 nM bsAbs or tsAb. (B) Quantification of CD8+ T cell activation with bsAbs or tsAbs by flow cytometry staining for CD25. The data are presented as the means ± SD, and statistical significance was calculated using Newman Keuls multiple comparison test: *P < 0.05, **P < 0.01, and ***P < 0.001; ns, not significant (≥0.05).

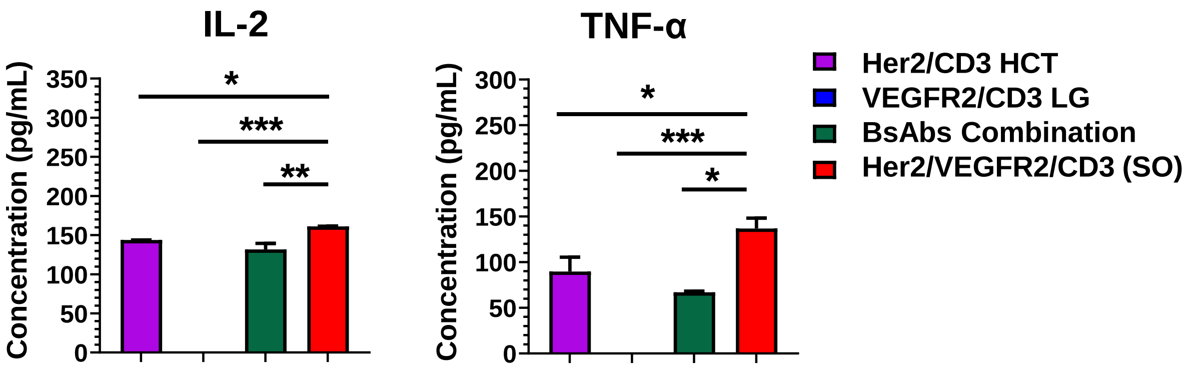

Supplementary Figure S20. Quantification of cytokine levels in the culture of PC3 cells and T cells in the presence of bsAbs and tsAb. IL-2 and TNF-α release was measured by ELISA kit from T cells cultured with PC3 cells (E: T = 20: 1) in the presence of 100pM bsAbs and tsAb. The data are presented as the means ± SD, and statistical significance was calculated using Newman Keuls multiple comparison test: *P < 0.05, **P < 0.01, and ***P < 0.001; ns, not significant (≥0.05).

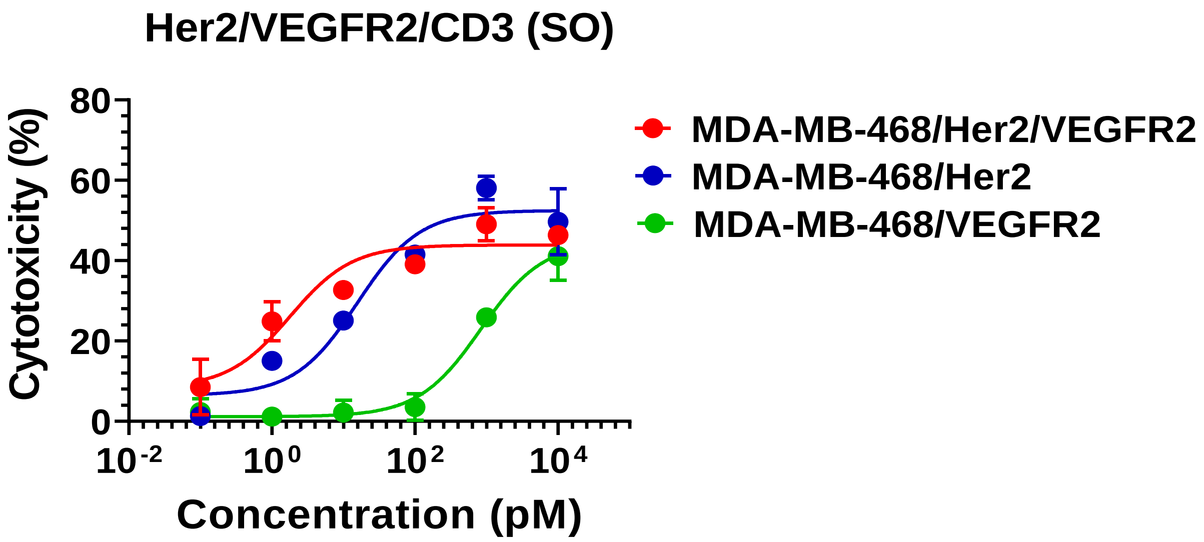

Supplementary Figure S21. Cytotoxicity of Her2/VEGFR2/CD3 (SO) redirecting T cells against different Her2- and/or VEGFR2-positive cancer cells. The cytotoxicity assays were performed with an E: T ratio of = 20:1 and after 24 h incubation. The cytotoxicity was assessed by LDH release. The data are presented as the means ± SD.

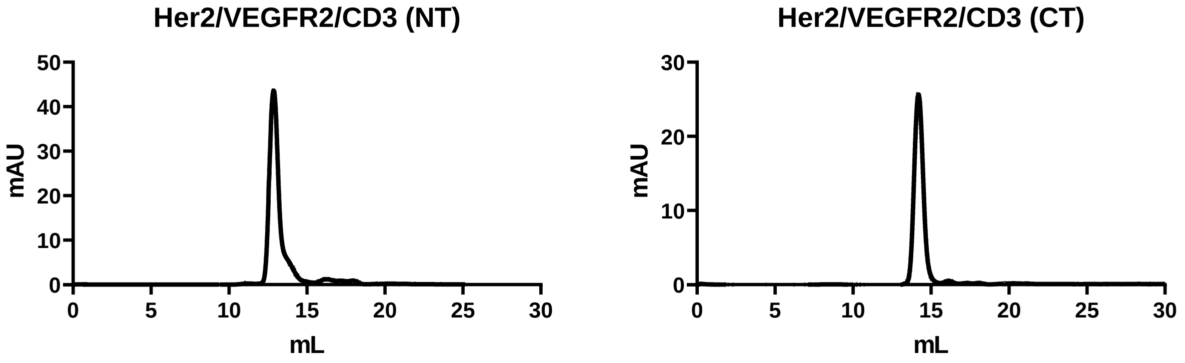

Supplementary Figure S22. Gel-filtration (Superdex 200) profiles of Her2/VEGFR2/CD3 (NT) and (CT) tsAbs.

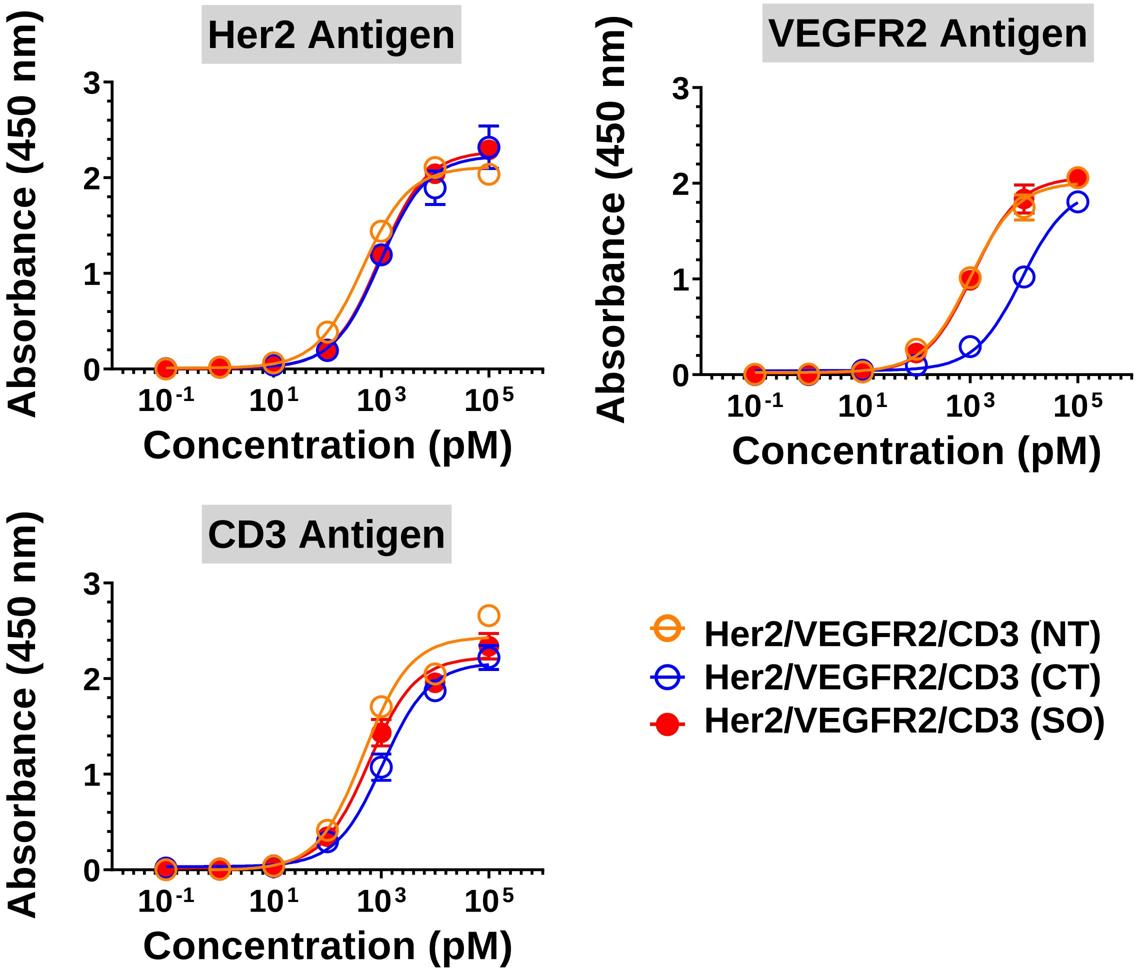

Supplementary Figure S23. Binding activities of different Her2/VEGFR2/CD3 tsAbs to human Her2-, VEGFR2- or CD3-extracellular domains by ELISA. Panels show different antigen-coated plates incubated with titrations of different tsAbs. The data are presented as the means ± SD.

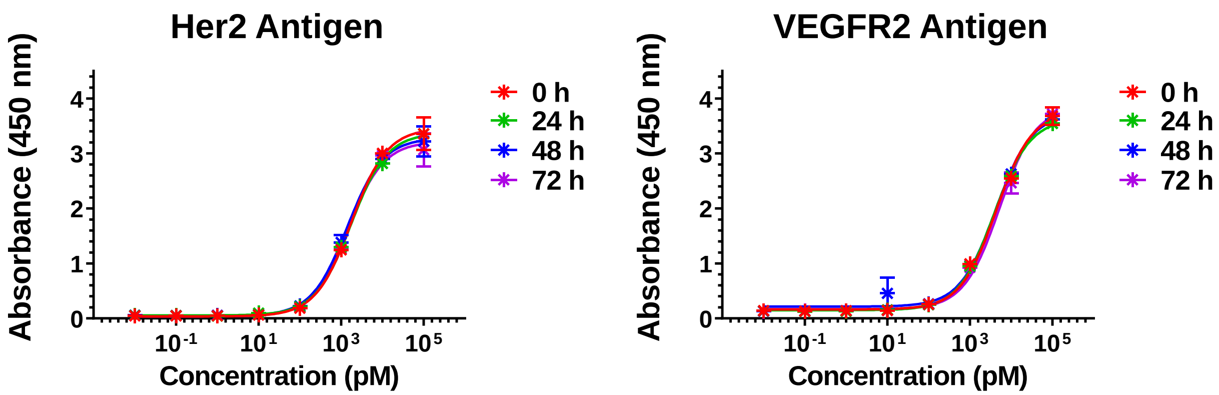

Supplementary Figure S24. Functional stability analysis of Her2/VEGFR2/CD3 (SO) by Her2- or VEGFR2-based ELISA. The proteins were incubated in human plasma at 37ºC for up to 72h, and then binding activities of different tsAb samples were tested by ELISA to human Her2- or VEGFR2-extracellular domains. The data are presented as the means ± SD.

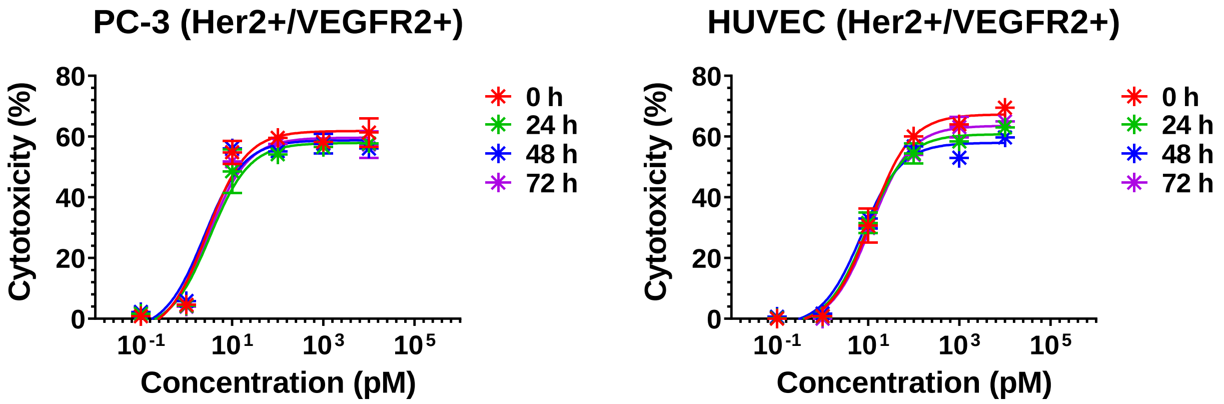

Supplementary Figure. S25. Functional stability analysis of Her2/VEGFR2/CD3 (SO) by T cell-mediated cytotoxicity on PC3 and HUVECs. The proteins were incubated in human plasma at 37ºC for up to 72h, and then the cytotoxicity assays were performed with an E: T ratio of = 10: 1 and after 24 h incubation. The cytotoxicity was assessed by LDH release. The data are presented as the means ± SD.

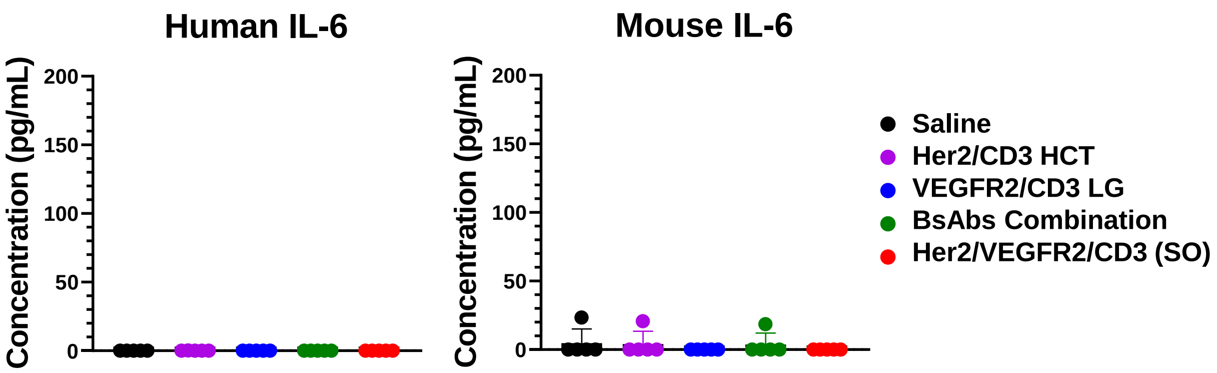

Supplementary Figure. S26. Quantification of human and mouse IL-6 levels 24 h after antibody treatment (n=5). Human and mouse IL-6 amounts were measured by ELISA kit from the serum of mice treated with different antibody fusions. The data are presented as the means ± SDs.

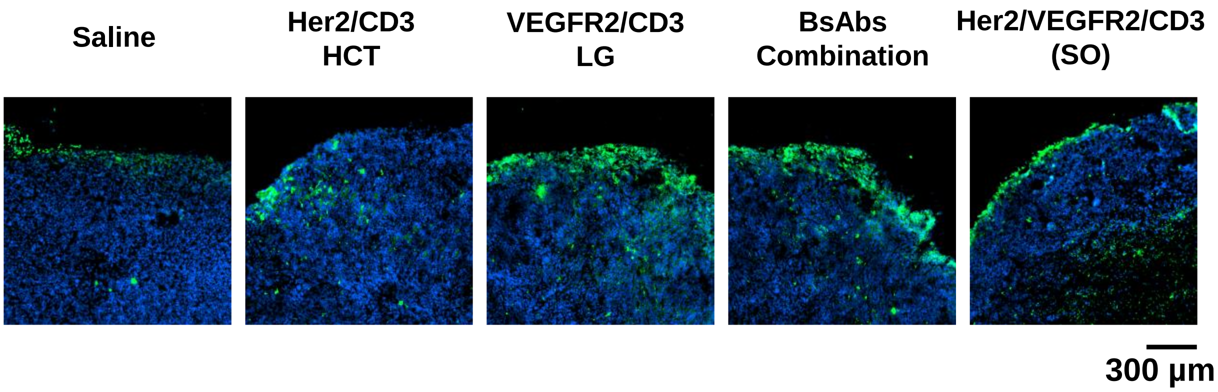

Supplementary Figure. S27. Immunofluorescence staining of tumor samples from the mice treated with saline, bsAb alone or the combination, or Her2/VEGFR2/CD3 (SO). The animals were sacrificed and frozen tumor sections were prepared and detected with anti-human CD3 antibody (green). DAPI (blue) was used for DNA staining.

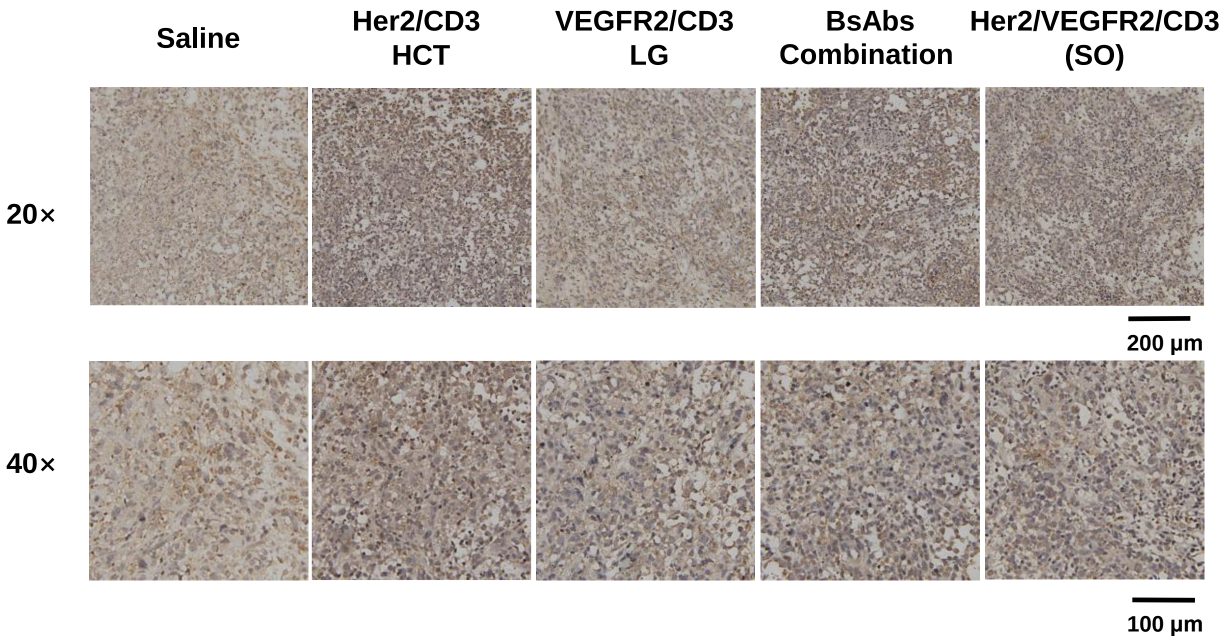

Supplementary Figure. S28. Immunohistochemistry analysis of Her2 expression of tumor samples from the mice treated with saline, bsAb alone or the combination, or Her2/VEGFR2/CD3 (SO). The animals were sacrificed and the tumor sections were fixed in 4% formaldehyde and embedded in paraffin. Immunohistochemistry was performed with antibodies against human Her2. Shown are representative images taken at × 20 and × 40 resolution.

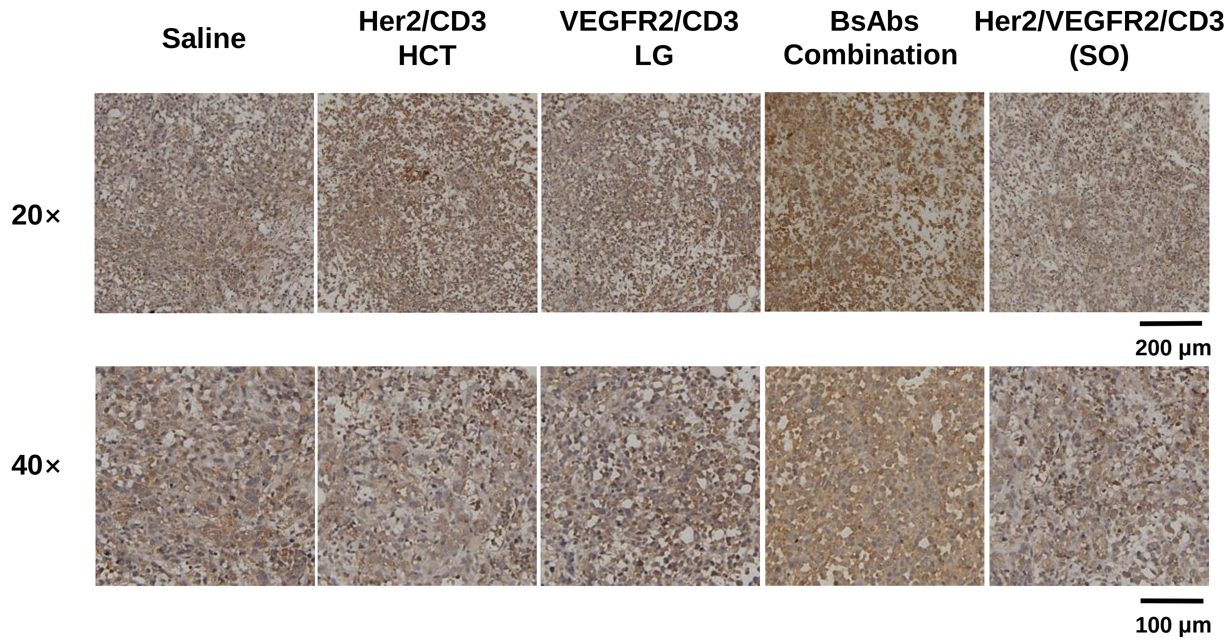

Supplementary Figure. S29. Immunohistochemistry analysis of VEGFR2 expression of tumor samples from the mice treated with saline, bsAb alone or the combination, or Her2/VEGFR2/CD3 (SO). The animals were sacrificed and the tumor sections were fixed in 4% formaldehyde and embedded in paraffin. Immunohistochemistry was performed with antibodies against human VEGFR2. Shown are representative images taken at × 20 and × 40 resolution.

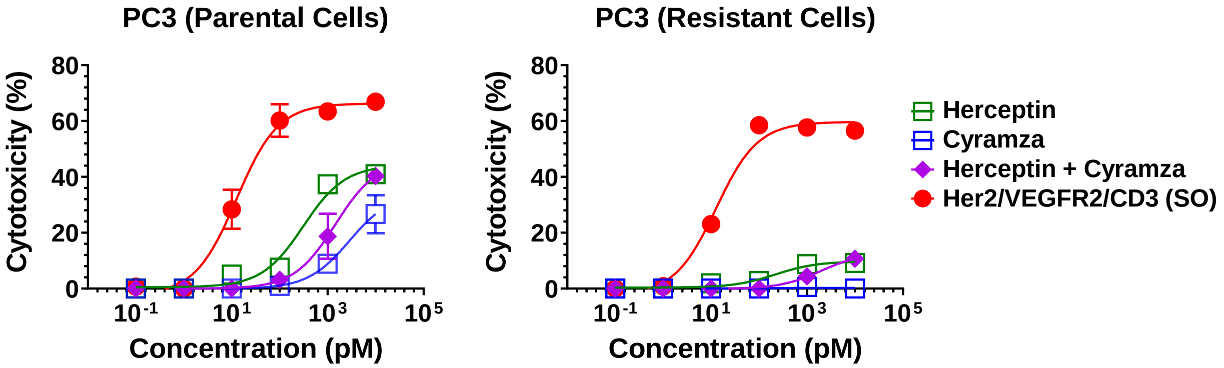

Supplementary Figure S30. Cytotoxicity of traditional antibodies and tsAbs against PC3 parental and resistant cells. Twenty-four-hour cytotoxicity assays were performed with human PBMCs and indicated target cells at 10: 1 ratio in the presence of different concentrations of Herceptin, Cyramza or their combination in comparison with tsAb.

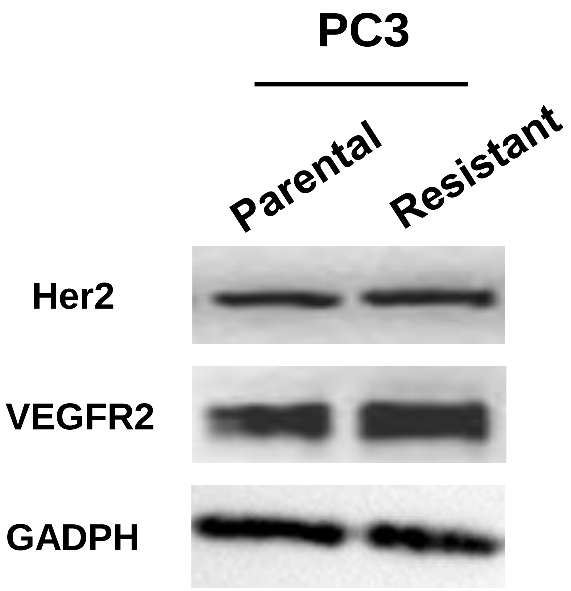

Supplementary Figure S31. Western blot analysis of Her2 and VEGFR2 expression in PC3 parental and resistant cells. The PC3 resistant cells were induced and cultured in the presence of 100nM of Herceptin and Cyramza.

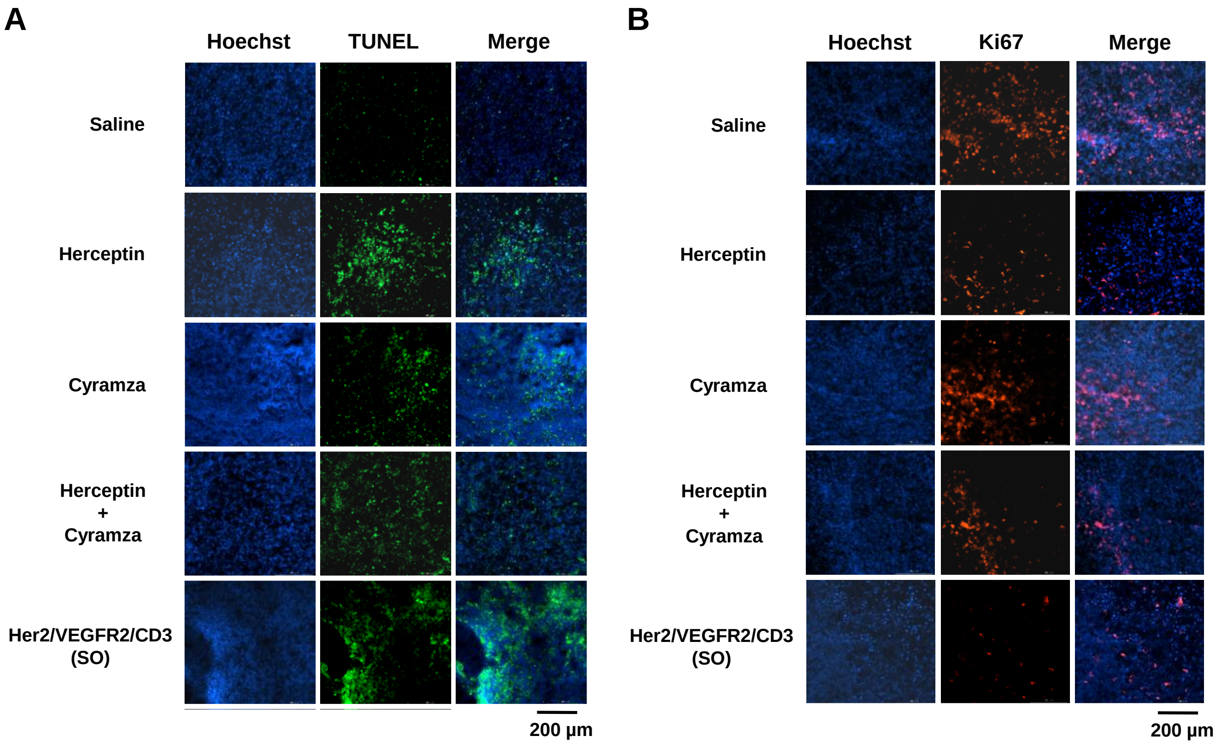

Supplementary Figure S32. Immunofluorescence staining of PC3-parental tumor samples. (A) Twenty-four hours after treatment, the animals were sacrificed, frozen tumor sections were prepared, and apoptosis was detected in the tumor tissue by TUNEL assay. DAPI was used for DNA staining. (B) Detection of cell proliferation in tumor samples by Ki67 staining.

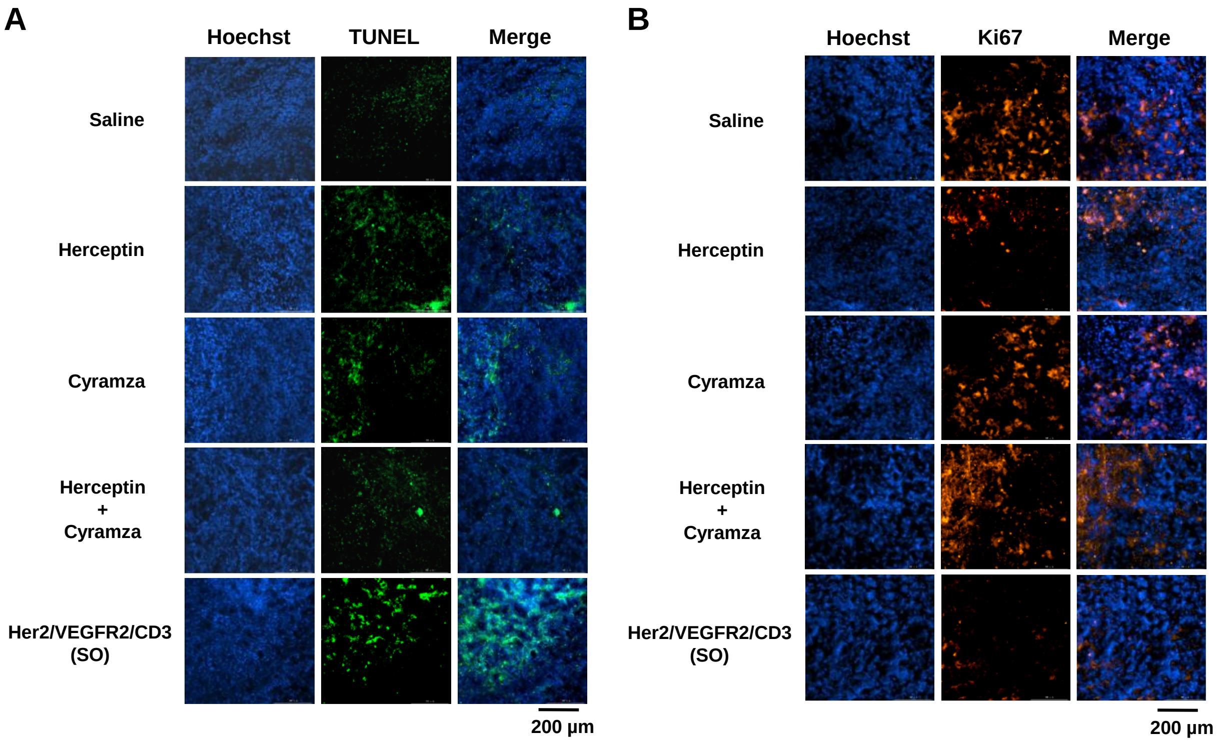

Supplementary Figure S33. Immunofluorescence staining of PC3-resistent tumor samples. (A) Twenty-four hours after treatment, the animals were sacrificed, frozen tumor sections were prepared, and apoptosis was detected in the tumor tissue by TUNEL assay. DAPI was used for DNA staining. (B) Detection of cell proliferation in tumor samples by Ki67 staining.

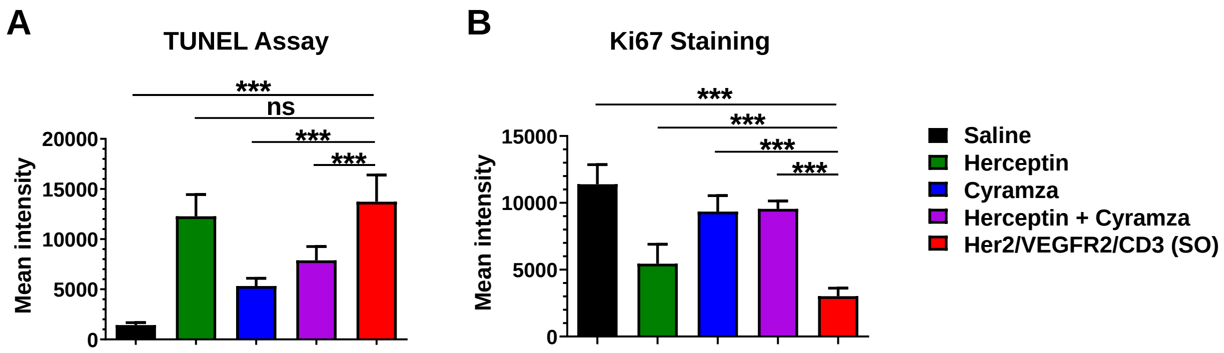

Supplementary Figure S34. Quantification of the apoptotic cell death (A) and cell proliferation (B) based on the fluorescent intensity of PC3 parental tumor samples detected by TUNEL assay. The data are presented as the means ± SD (n=15), and statistical significance was calculated using Newman Keuls multiple comparison test: *P < 0.05, **P < 0.01, and ***P < 0.001; ns, not significant (≥0.05).

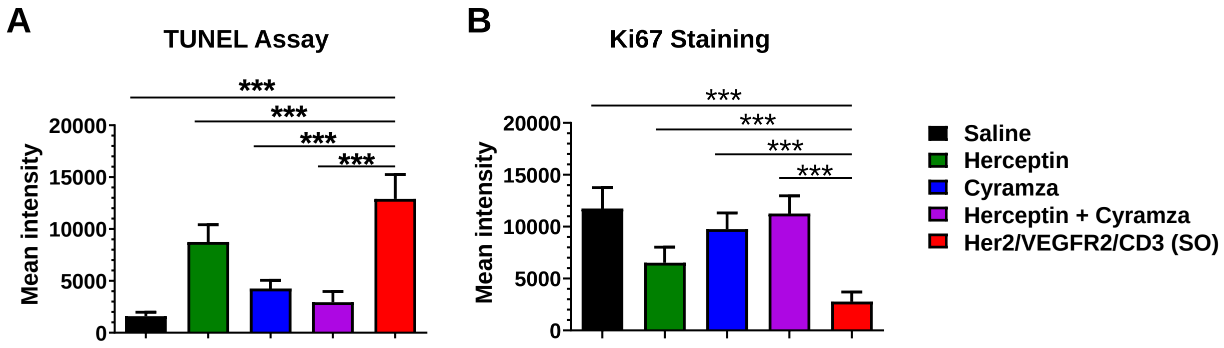

Supplementary Figure S35. Quantification of the apoptotic cell death (A) and cell proliferation (B) based on the fluorescent intensity of PC3 resistant tumor samples detected by TUNEL assay. The data are presented as the means ± SD (n=15), and statistical significance was calculated using Newman Keuls multiple comparison test: *P < 0.05, **P < 0.01, and ***P < 0.001; ns, not significant (≥0.05).

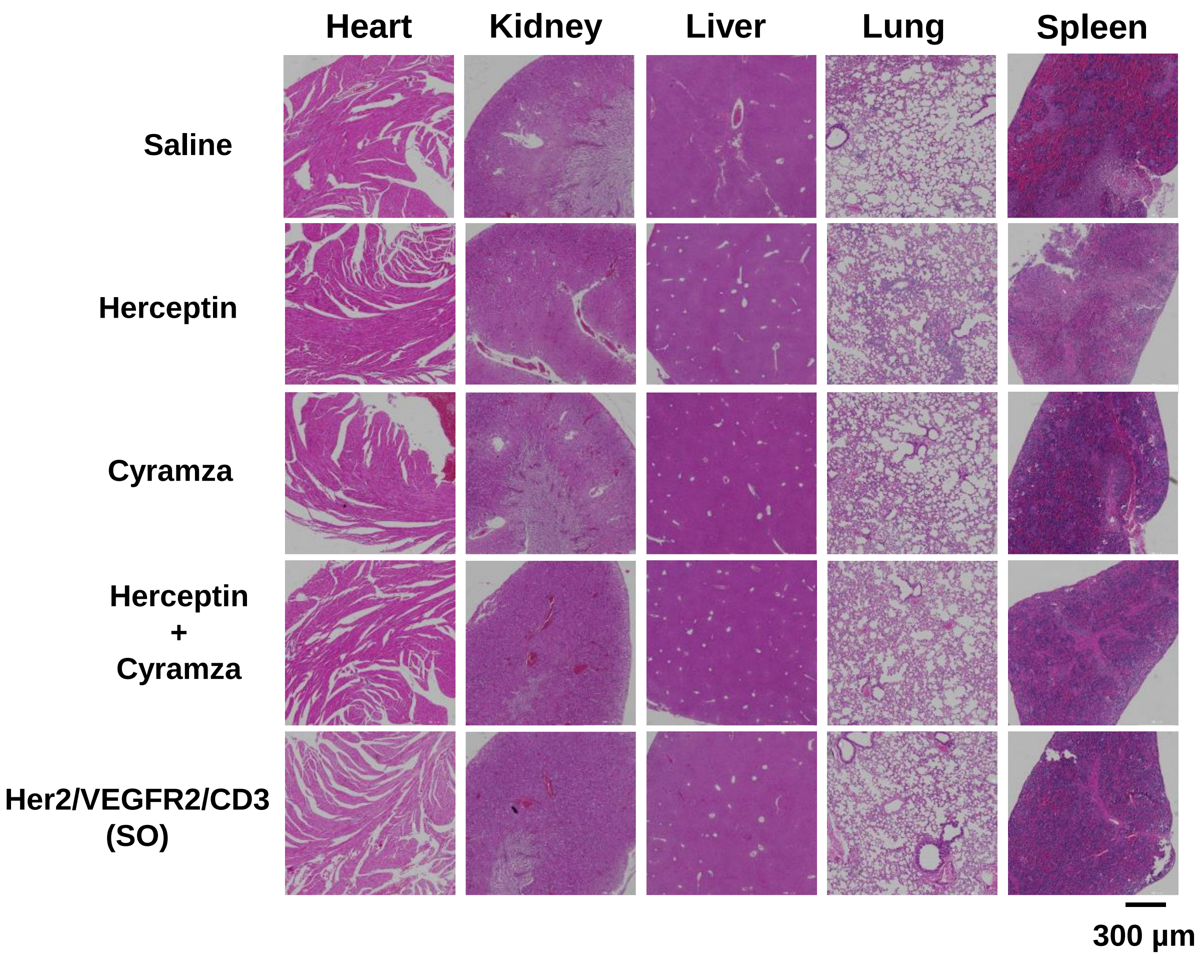

Supplementary Figure S36. Histology images of tissue samples with H&E staining after the treatment of the mice bearing PC3 parental tumors.

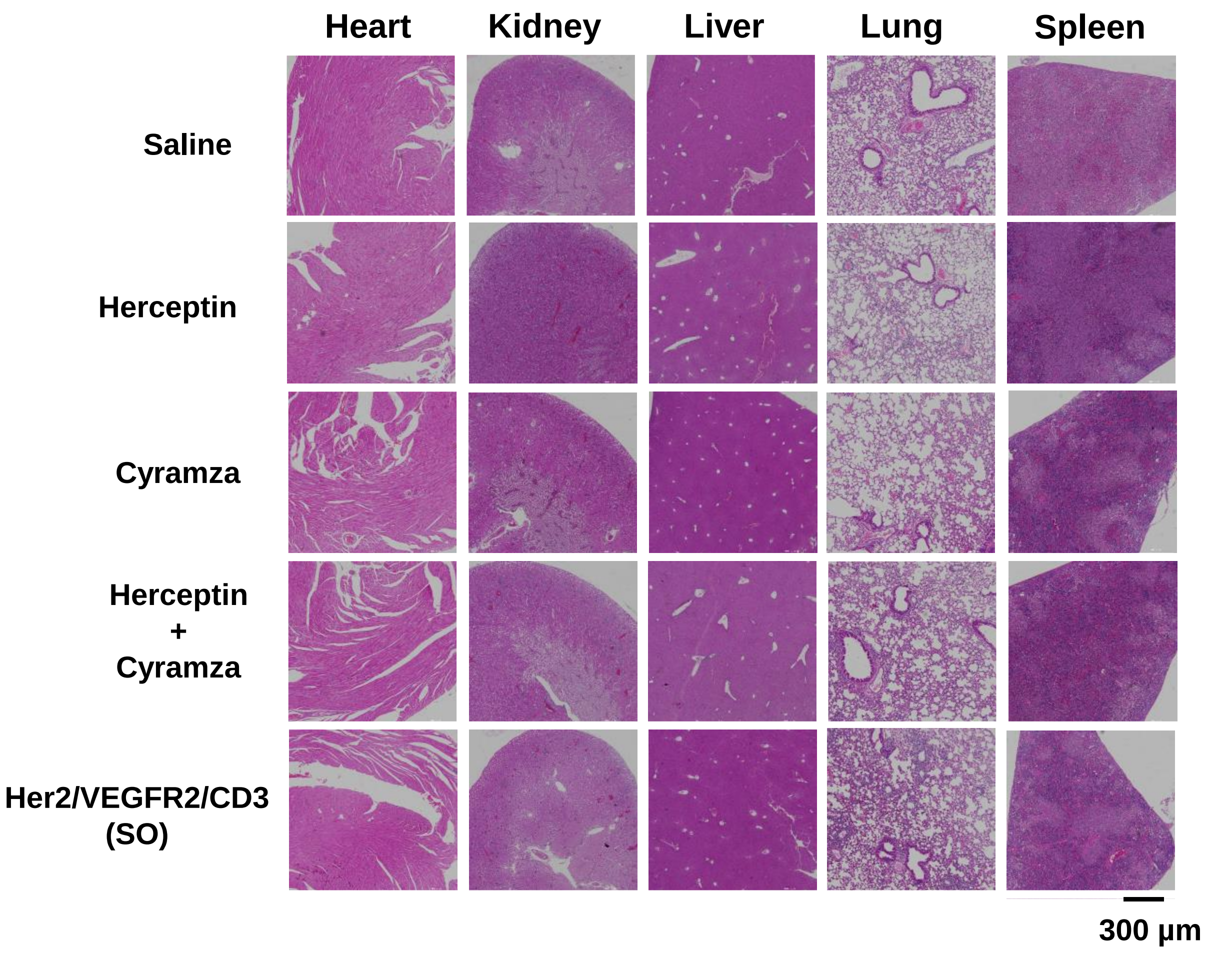

Supplementary Figure S37. Histology images of tissue samples with H&E staining after the treatment of the mice bearing PC3 resistant tumors.
